# Patient survival and tumor characteristics associated with *CHEK2*:p.I157T – findings from the Breast Cancer Association Consortium

**DOI:** 10.1186/s13058-016-0758-5

**Published:** 2016-10-03

**Authors:** Taru A. Muranen, Carl Blomqvist, Thilo Dörk, Anna Jakubowska, Päivi Heikkilä, Rainer Fagerholm, Dario Greco, Kristiina Aittomäki, Stig E. Bojesen, Mitul Shah, Alison M. Dunning, Valerie Rhenius, Per Hall, Kamila Czene, Judith S. Brand, Hatef Darabi, Jenny Chang-Claude, Anja Rudolph, Børge G. Nordestgaard, Fergus J. Couch, Steven N. Hart, Jonine Figueroa, Montserrat García-Closas, Peter A. Fasching, Matthias W. Beckmann, Jingmei Li, Jianjun Liu, Irene L. Andrulis, Robert Winqvist, Katri Pylkäs, Arto Mannermaa, Vesa Kataja, Annika Lindblom, Sara Margolin, Jan Lubinski, Natalia Dubrowinskaja, Manjeet K. Bolla, Joe Dennis, Kyriaki Michailidou, Qin Wang, Douglas F. Easton, Paul D. P. Pharoah, Marjanka K. Schmidt, Heli Nevanlinna

**Affiliations:** 1 Department of Obstetrics and Gynecology, University of Helsinki and Helsinki University Hospital, P.O. Box 700, 00029 HUS Helsinki, Finland; 2 Department of Oncology, University of Helsinki and Helsinki University Hospital, Helsinki, Finland; 3 Department of Clinical Genetics, University of Helsinki and Helsinki University Hospital, Helsinki, Finland; 4 Department of Pathology, University of Helsinki and Helsinki University Hospital, Helsinki, Finland; 5 Gynaecology Research Unit, Hannover Medical School, Hannover, Germany; 6 Department of Genetics and Pathology, Pomeranian Medical University, Szczecin, Poland; 7 Unit of Systems Toxicology, Finnish Institute of Occupational Health, Helsinki, Finland; 8 Faculty of Health and Medical Sciences, University of Copenhagen, Copenhagen, Denmark; 9 Copenhagen General Population Study, Herlev Hospital, Copenhagen University Hospital, Herlev, Denmark; 10 Department of Clinical Biochemistry, Herlev Hospital, Copenhagen University Hospital, Herlev, Denmark; 11 Centre for Cancer Genetic Epidemiology, Department of Oncology, University of Cambridge, Cambridge, UK; 12 Centre for Cancer Genetic Epidemiology, Department of Public Health and Primary Care, University of Cambridge, Cambridge, UK; 13 Department of Medical Epidemiology and Biostatistics, Karolinska Institutet, Stockholm, Sweden; 14 Department of Oncology - Pathology, Karolinska Institutet, Stockholm, Sweden; 15 Division of Cancer Epidemiology, German Cancer Research Center (DKFZ), Heidelberg, Germany; 16 University Cancer Center Hamburg (UCCH), University Medical Center Hamburg-Eppendorf, Hamburg, Germany; 17 Department of Laboratory Medicine and Pathology Mayo Clinic, Rochester, MN USA; 18 Department of Health Sciences Research, Mayo Clinic, Rochester, MN USA; 19 Division of Cancer Epidemiology and Genetics, National Cancer Institute, Rockville, MD USA; 20 Division of Genetics and Epidemiology, The Institute of Cancer Research, London, UK; 21 Department of Gynaecology and Obstetrics, University Hospital Erlangen, Friedrich-Alexander University Erlangen-Nuremberg, Comprehensive Cancer Center Erlangen-EMN, Erlangen, Germany; 22 Department of Medicine, Division of Hematology and Oncology, David Geffen School of Medicine, University of California at Los Angeles, Los Angeles, CA USA; 23 Human Genetics Division, Genome Institute of Singapore, Singapore, Singapore; 24 Lunenfeld-Tanenbaum Research Institute of Mount Sinai Hospital, Toronto, ON Canada; 25 Department of Molecular Genetics, University of Toronto, Toronto, ON Canada; 26 Laboratory of Cancer Genetics and Tumor Biology, Northern Finland Laboratory Centre NordLab, Oulu, Finland; 27 Laboratory of Cancer Genetics and Tumor Biology, Cancer and Translational Medicine Research Unit and Biocenter Oulu, University of Oulu, Oulu, Finland; 28 Cancer Center, Kuopio University Hospital, Kuopio, Finland; 29 Imaging Center, Department of Clinical Pathology, Kuopio University Hospital, Kuopio, Finland; 30 Institute of Clinical Medicine, Pathology and Forensic Medicine, University of Eastern Finland, Kuopio, Finland; 31 Central Finland Hospital District, Jyväskylä Central Hospital, Jyväskylä, Finland; 32 Department of Electron Microscopy/Molecular Pathology, The Cyprus Institute of Neurology and Genetics, Nicosia, Cyprus; 33 Netherlands Cancer Institute, Antoni van Leeuwenhoek Hospital, Amsterdam, The Netherlands

**Keywords:** Breast cancer, CHEK2, CHK2, I157T, 1100delC, Survival, Pathology, Gene expression

## Abstract

**Background:**

P.I157T is a *CHEK2* missense mutation associated with a modest increase in breast cancer risk. Previously, another *CHEK2* mutation, the protein truncating c.1100delC has been associated with poor prognosis of breast cancer patients. Here, we have investigated patient survival and characteristics of breast tumors of germ line p.I157T carriers.

**Methods:**

We included in the analyses 26,801 European female breast cancer patients from 15 studies participating in the Breast Cancer Association Consortium. We analyzed the association between p.I157T and the clinico-pathological breast cancer characteristics by comparing the p.I157T carrier tumors to non-carrier and c.1100delC carrier tumors. Similarly, we investigated the p.I157T associated risk of early death, breast cancer-associated death, distant metastasis, locoregional relapse and second breast cancer using Cox proportional hazards models.

Additionally, we explored the p.I157T-associated genomic gene expression profile using data from breast tumors of 183 Finnish female breast cancer patients (ten p.I157T carriers) (GEO: GSE24450). Differential gene expression analysis was performed using a moderated *t* test. Functional enrichment was investigated using the DAVID functional annotation tool and gene set enrichment analysis (GSEA). The tumors were classified into molecular subtypes according to the St Gallen 2013 criteria and the PAM50 gene expression signature.

**Results:**

P.I157T was not associated with increased risk of early death, breast cancer-associated death or distant metastasis relapse, and there was a significant difference in prognosis associated with the two *CHEK2* mutations, p.I157T and c.1100delC. Furthermore, p.I157T was associated with lobular histological type and clinico-pathological markers of good prognosis, such as ER and PR expression, low TP53 expression and low grade. Gene expression analysis suggested luminal A to be the most common subtype for p.I157T carriers and CDH1 (cadherin 1) target genes to be significantly enriched among genes, whose expression differed between p.I157T and non-carrier tumors.

**Conclusions:**

Our analyses suggest that there are fundamental differences in breast tumors of *CHEK2*:p.I157T and c.1100delC carriers. The poor prognosis associated with c.1100delC cannot be generalized to other *CHEK2* mutations.

**Electronic supplementary material:**

The online version of this article (doi:10.1186/s13058-016-0758-5) contains supplementary material, which is available to authorized users.

## Background

Checkpoint kinase 2 (*CHEK2*) is a moderate penetrance breast cancer risk gene. The two most frequent *CHEK2* mutations in European populations are p.I157T and c.1100delC. Truncating *CHEK2* founder mutations (c.1100delC, IVS2 + 1G > A, del5395) confer a higher than twofold increase in the risk of breast cancer [1–3], whereas p.I157T (c.470 T > C, rs17879961), a *CHEK2* missense mutation is associated with a milder, 1.4-fold elevation in the risk [4]. The c.1100delC carrier frequency is highest in the Netherlands and in Finland (over 1 %), the other two truncating founder mutations are found mainly in Poland [3], and p.I157T is most frequent in Finland and in Poland (around 5 %) [5]. Additionally, dozens of rare *CHEK2* missense mutations have been found in breast cancer patients, but their contribution to disease risk is minor on a population level and causative role in disease development probably varies greatly [6–8].

The consequences of c.1100delC and p.I157T differ on a molecular level, but both have been shown to severely interfere with the CHEK2 protein activity. C.1100delC is a loss-of-function mutation that induces a premature termination codon in the kinase domain in exon 10 (ter381) leading to a nonsense-mediated mRNA decay, which reduces both mutated and overall *CHEK2* mRNA level [9, 10]. C.1100delC truncates CHEK2 protein’s C-terminal kinase domain. The truncated protein is unstable and practically undetectable in mutation carrier cells [9]. Isoleucine 157 (p.I157T) is required for several van der Waals interactions at the interface of forkhead-associated (FHA) and kinase domains of dimerizing CHEK2 peptide chains. Its replacement to threonine (p.I157T) has been shown to interfere with these interactions and to severely impede the CHEK2 homodimerization required for its activation [11]. Furthermore, ectopic expression of human *CHEK2*:p.I157T failed a *rad53/sml* complementation assay in yeast suggesting an impaired protein function [6]. Thus, p.I157T possibly disturbs CHEK2 function by competing with the wild-type protein in dimer formation in heterozygous cells in a dominant negative manner [4].

Since both p.I157T and c.1100delC cause increased risk of breast cancer and compromise the activity of the CHEK2 protein, the question remains whether their effects on patient prognosis would be proportional to their risk effects and how similar the breast cancer phenotypes associated with the mutations would be. C.1100delC is associated with bilateral disease and estrogen receptor (ER)-positive tumors [12–14]. However, although tumors from p.I157T carriers are also predominantly ER-positive [15], tumors from p.I157T and c.1100delC carriers are associated with phenotypically different types of breast cancer. The lobular histological type is overrepresented among p.I157T mutation carrier tumors [16], whereas the c.1100delC carrier tumors are typically ductal [13, 14].

We have previously reported *CHEK2*:c.1100delC heterozygosity to be associated with reduced overall and disease-free survival as well as with increased risk of breast cancer-specific death in a Breast Cancer Association Consortium (BCAC) data set combining mutation carriers from multiple European populations [17]. Here, we report a study investigating thoroughly the prognostic associations of *CHEK2*:p.I157T as well as pathologic characteristics and genomic gene expression profiles of breast tumors from carriers of germ line p.I157T.

## Methods

### Study subjects for survival and pathology analyses

We included in the analyses female invasive breast cancer patients of European ancestry with a first invasive primary breast cancer enrolled in 15 studies participating in the Breast Cancer Association Consortium (BCAC) (Additional file 1: Table S1). In order to be able to stratify the analyses by study, only BCAC studies providing genotype and survival data of about ten *CHEK2*:p.I157T carriers were included in the analyses (Additional file 1: Table S2). Altogether, the data set consisted of 26,801 study subjects, of which 590 carried germ line p.I157T and 271 carried c.1100delC mutations (Table 1). Individuals carrying both mutations were excluded from the analyses (*n* = 4).

**Table 1:** Tumor characteristics of the BCAC study subjects

		Non-carriers	p.I157T carriers	c.1100delC carriers	*p* value (I157T/nc)	*p* value (I157T/1100delC)	*p* value (1100delC/nc)
ER	Negative	4595	85	26	0.00046	0.61	0.0015
		*20.2 %*	*15.5 %*	*11.2 %*			
	Positive	18,179	462	207			
		*79.8 %*	*84.5 %*	*88.8 %*			
	Missing	3166	43	38			
		*12.2 %*	*7.3 %*	*14.0 %*			
PR	Negative	6397	147	44	0.0034	0.90	0.0045
		*32.7 %*	*28.5 %*	*23.3 %*			
	Positive	13,173	368	145			
		*67.3 %*	*71.5 %*	*76.7 %*			
	Missing	6370	75	82			
		*24.6 %*	*12.7 %*	*30.3 %*			
Her2	Negative	8220	231	95	0.68	0.10	0.24
		*84.7 %*	*83.7 %*	*81.9 %*			
	Positive	1483	45	21			
		*15.3 %*	*16.3 %*	*18.1 %*			
	Missing	16,237	314	155			
		*62.6 %*	*53.2 %*	*57.2 %*			
EGFR	Negative	3841	122	62	0.21	0.26	0.034
		*89.6 %*	*90.4 %*	*96.9 %*			
	Positive	448	13	2			
		*10.4 %*	*9.6 %*	*3.1 %*			
	Missing	21,651	455	207			
		*83.5 %*	*77.1 %*	*76.4 %*			
CK5/6	Negative	4734	143	80	0.30	0.29	0.19
		*87.9 %*	*88.3 %*	*92.0 %*			
	Positive	652	19	7			
		*12.1 %*	*11.7 %*	*8.0 %*			
	Missing	20,554	428	184			
		*79.2 %*	*72.5 %*	*67.9 %*			
TP53	Negative	3755	144	88	0.00048	0.21	0.16
		*81.6 %*	*90.6 %*	*86.3 %*			
	Positive	847	15	14			
		*18.4 %*	*9.4 %*	*13.7 %*			
	Missing	21,338	431	169			
		*82.3 %*	*73.1 %*	*62.4 %*			
Tumor size (ordinal)	<20 mm	14,949	340	149	0.29	0.33	0.83
		*65.6 %*	*65.6 %*	*62.6 %*			
	20–50 mm	6953	162	82			
		*30.5 %*	*31.3 %*	*34.5 %*			
	>50 mm	876	16	7			
		*3.8 %*	*3.1 %*	*2.9 %*			
	Missing	3162	72	33			
		*12.2 %*	*12.2 %*	*12.2 %*			
Lymph node status	Negative	13,144	320	125	0.94	0.91	0.64
		*62.0 %*	*60.4 %*	*57.6 %*			
	Positive	8070	210	92			
		*38.0 %*	*39.6 %*	*42.4 %*			
	Missing	4726	60	54			
		*18.2 %*	*10.2 %*	*19.9 %*			
Grade (ordinal)	1	4916	132	38	0.00023	0.0030	0.38
		*22.5 %*	*26.7 %*	*17.4 %*			
	2	10,817	266	127			
		*49.6 %*	*53.7 %*	*58.0 %*			
	3	6089	97	54			
		*27.9 %*	*19.6 %*	*24.7 %*			
	Missing	4118	95	52			
		*15.9 %*	*16.1 %*	*19.2 %*			
Histological type	Ductal	14,133	273	193	0.0044^*^	0.0010^#^	0.67^#^
		*72.7 %*	*60.0 %*	*76.9 %*			
	Lobular	2966	100	36			
		*15.3 %*	*22.0 %*	*14.3 %*			
	Mixed *(ductal and lobular)*	455	26	4			
		*2.5 %*	*6.0 %*	*1.7 %*			
	Tubular	271	17	2			
		1.5 %	4.0 %	*0.7 %*			
	Medullary	177	4	3			
		*1.0 %*	*0.9 %*	*1.7 %*			
	Mucinous	213	9	3			
		*1.2 %*	*2.1 %*	*1.4 %*			
	Papillary	55	1	1			
		*0.3 %*	*0.2 %*	*1.8 %*			
	Missing†	7670	160	29			
		*29.6 %*	*27.1 %*	*10.7 %*			
Subtype‡	LumA (ER+, PR+, Her2-)	5415	164	72	0.00089	0.46	0.0087
		58.9 %	63.6 %	65.5 %			
	LumB (ER+, PR-, Her2- or ER+, Her2+)	1939	62	30			
		21.1 %	24.0 %	27.3 %			
	Basal (ER-, PR-, Her2-)	1306	16	4			
		14.2 %	6.2 %	3.6 %			
	Her2-positive (ER-, PR-, Her2+)	536	16	4			
		5.8 %	6.2 %	3.6 %			
	Missing	16,744	332	161			
		64.5 %	56.3 %	59.4 %			
AGE cat (ordinal)	50 or younger	6932	162	101	0.99	0.0037	0.0029
		*27.0 %*	*27.8 %*	*37.3 %*			
	Older than 50 and not more than 70	16,083	344	151			
		*62.6 %*	*59.0 %*	*55.7 %*			
	Older than 70	2674	77	19			
		*10.4 %*	*13.2 %*	*7.0 %*			
	Missing	251	7	0			
		*1.0 %*	*1.2 %*	*0.0 %*			
AGE	Mean	57.1	57.9	54.3	0.41	0.028	0.024
	St.dev.	10.8	11.1	11.0			
Total		25,940	590	271			

*BCAC* Breast Cancer Association Consortium, *ER* estrogen receptor, *PR* progesterone receptor, *Her2* human epidermal growth factor receptor 2, *EGFR* epidermal growth factor receptor, *CK5/6* cytokeratin 5/6, *TP53* tumor protein 53, *LumA* luminal A, *LumB* luminal B

^*^Categories Medullary, Mucinous and Papillary were combined for the Cochran-Mantel-Haenszel test

^#^Categories Mixed, Tubular, Medullary, Mucinous and Papillary were combined for the Cochran-Mantel-Haenszel test

^†^The “missing” category included also rare forms of breast cancer, which did not belong to the named categories: 1179 non-carriers, 25 p.I157T carriers and 9 c.1100delC carriers

^‡^Tumor subtypes are defined according to ER, PR and Her2 expression following the St Gallen 2013 guidelines [34]

Italics is used to indicate the proportion of study subjects in each category. E.G. 'ER-positive/all with known ER-status' or 'missing/all study subjects'

### Genotyping

*CHEK2*:p.I157T was first genotyped by independent studies using various methods including MassARRAY iPLEX Gold (Sequenom, San Diego, CA, USA), TaqMan (Applied Biosystems, Life Technologies, Carlsbad, CA, USA) and Fluidigm (Fluidigm, San Francisco, CA, USA) as listed in Additional file 1: Table S1. Quality control was implemented as follows: each study performed duplicate measurements of at least two samples from each sample plate and genotyped 93 CEPH control DNAs (HAPMAPPT01, Coriell Institute for Medical Research, Cambden, NJ, USA). If a study reported more than two discordant genotyping results of the CEPH DNAs, all genotype data from that study was excluded. Later, p.I157T was genotyped centrally using a custom Illumina iSelect genotyping array for the Collaborative Oncological Gene-environment Study (COGS) [18]. Discordant genotyping results were clarified with Sanger sequencing. *CHEK2*:c.1100delC was genotyped by independent studies using mainly TaqMan (Additional file 1: Table S1), as described earlier [17].

### Pathology analysis

Pathology data was collected from hospital records or from scientific projects within the individual studies, as described previously [19]. Additionally, the TP53 protein expression was measured by individual studies using immunohistochemical staining as described in Additional file 1: Table S3. The pathology data availability and mutation carrier frequencies varied between independent BCAC studies and therefore all analyses were stratified by study. Pathology analyses were performed using R environment for statistical computing version 3.0.2 [20] including packages vcdExtra [21] and meta [22]. Comparisons were made between *CHEK2* mutations carriers (heterozygous or homozygous) and non-carriers, for both p.I157T and c.1100delC, as well as between carriers of p.I157T and c.1100delC (Table 1). Associations between the mutations and clinico-pathological characteristics were tested with study-stratified Cochran-Mantel-Haenszel test (mantelhaen.test for categorical characteristics and CMHtest for ordinal characteristics). The category of missing data was not included in these comparisons. Differences in age at diagnosis were tested by meta-analysis of age distribution in independent studies using a random effects model (metacont).

### Survival analysis

Survival analyses were performed using the Cox regression [23] as implemented in Stata (Stata/SE 10.1 for Windows, StataCorp LP, College Station, TX, USA) comparing *CHEK2* mutation carriers and non-carriers, as described above. Study subjects were considered to become at risk at the time of their first invasive breast cancer diagnosis. The data did not consist entirely of incident cases. Therefore, in order to avoid bias caused by late enrollment, we implemented a method called left censoring, which has been proven to provide robust survival estimates for data, which includes also prevalent cases [24]. Survival analysis endpoints included death of any cause, breast cancer-associated death, distant metastasis relapse, locoregional relapse and second breast cancer. Patients were censored at the end of their follow-up period or at the latest 15 years after the initial breast cancer diagnosis in analyses of overall survival and second breast cancer, but at the latest 10 years in analyses of locoregional or distant relapse-free survival as well as in analyses of breast cancer-specific survival. Patients presenting with distant metastases at diagnosis were excluded from the analyses of locoregional relapse-free survival. All analyses were stratified by study.

In addition to univariate analyses, we performed multivariate analyses, which were stratified by study and age category (≤50 years; >50 and ≤70; >70), and adjusted for tumor grade (1, 2 or 3, ordinal), tumor size (1: maximum diameter less than or equal to 20 mm; 2: more than 20 mm and less than or equal to 50 mm; 3 over 50 mm, ordinal), tumor spread in axillary lymph nodes (0 = negative, 1 = positive) and progesterone receptor (PR) status (0 = negative, 1 = positive). ER was not included in the model, because of the non-linear relationship between tumor ER status and patient survival during the 10 years following the diagnosis; patients with ER-negative tumors have a higher risk of dying from breast cancer during the first 5 years after the diagnosis, but the difference in risk between ER-positive and ER-negative tumors levels out after that period [17, 25]. However, since several studies have reported an association between the two *CHEK2* mutations and ER-positive disease [12–14] (Table 1), we performed the survival analyses in a subgroup of patients with ER-positive tumors. Only cases with complete data on the pathological markers were included in the multivariate analyses. Univariate survival analyses were performed also in a subgroup of breast cancer patients with lobular tumors, because of the association between p.I157T and lobular breast cancer [15] (Table 1).

### Study subjects for gene expression analysis

Gene expression analyses were performed using a data set of 183 breast tumors from the Helsinki University Hospital (GEO: GSE24450). As described previously, the data set consisted of total RNA samples from 151 tumors from unselected cohorts of breast cancer patients and 32 tumors from additional familial cases hybridized on Illumina HumanHT-12 v3 Expression BeadChips (Illumina Inc., San Diego, CA, USA) [10, 26]. The p.I157T carrier status was defined from peripheral blood samples as described earlier for the BCAC study ‘HEBCS’ (Additional file 1: Table S1). Ten patients were germ line p.I157T carriers and 162 were non-carriers, of which six carried germ line c.1100delC. The c.1100delC carrier tumors were included in the analyses as non-I157T carriers. The p.I157T genotype information was not available for 11 study subjects. These were included in the molecular subtype analysis, but not in differential gene expression or gene set enrichment analysis. The clinico-pathologic characteristics of the 183 tumors are provided in Additional file 1: Table S4.

### Gene expression analysis

Gene expression data quality control and quantile normalization was performed in the Bioconductor [27] as described earlier [26]. Data analyses were performed in R version 3.0.2 and Bioconductor packages genefu [28], limma [29, 30] and geneplotter [31]. Probes not mapping to any current Entrez Gene entities (GRCh38.p2) were excluded, resulting in a filtered data set of 20,145 genes.

For determining the intrinsic molecular subtypes, expression data of the fifty PAM50 signature genes was extracted from the filtered data set, median centered and standardized per gene by dividing with the standard deviation of the gene’s expression values. Intrinsic subtypes were defined by Pearson correlation between tumors and the luminal A, luminal B, human epidermal growth factor receptor 2 (Her2)-enriched, basal-like and normal-like centroids as implemented in the genefu package [28, 32]. Hierarchical clustering was performed using the Ward’s method [33]. As a comparison to the subtype classification by gene expression, we used the surrogate clinico-pathologic markers to define the subtypes following the St Gallen 2013 criteria (luminal A: ER+, PR+, Her2-, Ki-67-; luminal B (three marker combinations): ER+, PR-, Her2- or ER+, Her2-, Ki-67+ or ER+, Her2+; basal: ER-, PR-, Her2-; Her2 overexpressing: ER-, PR-, Her2+) [34].

For analysis of differential gene expression the data was filtered by including only genes with highest variation in expression levels over the entire data set (st. dev. ≥ 0.75, 1852 genes). The samples from p.I157T carriers were compared to samples from non-carriers with a moderated *t* test adjusting for ER, tumor protein 53 (TP53) and Ki-67 protein expression (positive/negative), tumor grade (1, 2, 3, ordinal) as well as histological type (lobular/other). The adjusting covariates were selected from features tabulated in Additional file 1: Table S4 as the most significant factors (*p* < 0.001) explaining variation in the expression of the 1852 genes as summarized by the first four principal components. Additionally, lobular histologic type was included to avoid bias caused by the association between the p.I157T and lobular type. Data on at least one of the adjusting variables was missing for 12 tumor samples and thus the differential gene expression and gene set enrichment analyses were performed with a set of 160 (ten p.I157T and 150 non-carrier) tumor samples and 1852 genes. Genes with *p* values below 0.01 were considered to be associated with p.I157T. These were included in a functional enrichment analysis performed using the DAVID functional annotation tool [35]. Functional annotations with Benjamini-Hochberg [36] corrected *p* values below 0.01 were considered to be significantly enriched.

Gene set enrichment analysis (GSEA) was performed using a java application available at http://software.broadinstitute.org/gsea following the instructions of the user guide [37]. For the GSEA analysis, the 1852 genes were ranked according to a score calculated as the product of log_2_(fold change) and log_10_(*p* value) from comparisons of p.I157T carrier and non-carrier tumors as described above. All gene sets available at the Molecular Signatures Database (MSigDB) v5.0 [38] were included in the analyses. The *p* values were corrected for false discovery rate for all other gene sets but the gene sets originating from single publications (‘CGP: chemical and genetic perturbations’ database), which were corrected for the family-wise error rate. Gene sets with corrected *p* value below 0.05 were considered to be significantly enriched in the p.I157T carrier tumors.

## Results and discussion

Our findings from extensive analyses of breast tumor phenotypes and patient survival underline a fundamental difference in breast cancers of the carriers of two *CHEK2* mutations, p.I157T and c.1100delC. Significant differences were found in tumor grade and histopathological type as well as in patient survival of p.I157T and c.1100delC carriers, whereas no difference was seen in tumor subtypes: ER+, PR+, Her2- disease was the most common type for carriers of both mutations.

### Association of p.I157T with clinico-pathological markers

In our analyses p.I157T was associated with low grade as well as several other markers, which have previously been associated with good prognosis (Table 1). Our analyses confirmed the previously reported associations between p.I157T and ER-positive or lobular breast cancer [15]. Also mixed (ductal and lobular) and tubular histological types were more frequent in p.I157T carrier tumors. Both of ER-positive and lobular tumor types are associated with good short-term prognosis, but increased risk of disease progression in the long run [25, 39]. Furthermore, p.I157T was associated with PR-positive and TP53-negative breast cancer. PR expression is a marker for good prognosis for ER-positive breast cancer and it has been suggested as a surrogate marker separating luminal A and luminal B subtypes according to immunohistochemical data [34, 40, 41]. TP53 immunohistochemical staining is considered to be indicative of somatic *TP53* mutations. Strong TP53 staining suggests the presence of stabilizing mutations (primarily missense), whereas absence of staining indicates typically a protein-truncating mutation (nonsense or frameshift), and weak staining a wild-type functional *TP53*. Both strong and completely negative TP53 staining have been associated with poor prognosis in comparison to weak staining [42–44]. The sensitivity of the assays used in this study did not enable differentiation between normal, low or absent TP53 expression. Therefore, we used binary classification of TP53 immunohistochemical data, the positive category corresponding to high expression (strong staining) and negative category to low expression (Additional file 1: Table S3). Noteworthy, the loss-of-function mutations associated with absent TP53 staining are relatively rare in breast cancer: these are seen in less than 5 % of all tumors [45, 46]. Therefore, it is likely that most of the tumors in the category ‘negative’ (Table 1) represented tumors with wild-type *TP53*. However, compromised CHEK2 function as a result of the p.I157T mutation could be another way for TP53 silencing as CHEK2 is among the key upstream activators of TP53 [5].

Like p.I157T, also c.1100delC was associated with ER-positive and PR-positive disease in our data set (Table 1). Furthermore, TP53-positive tumors were slightly less often observed in c.1100delC carriers than in non-carriers, even though the difference was not statistically significant. Significant differences in clinico-pathological features associated with the two *CHEK2* mutations were seen in grade and histological type, as the c.1100delC carrier tumors resembled more non-carrier tumors (Table 1).

### Breast cancer subtypes

We investigated the I1157T-associated molecular breast cancer subtypes by applying the St Gallen 2013 criteria for immunohistochemical markers [34] on the BCAC data set as well as St Gallen 2013 and the PAM50 classifier [32] on the gene expression data set of 183 breast tumors. The subtype classification of the BCAC study subjects relied on the available immunohistochemical markers, ER, PR and Her2. We found both p.I157T and c.1100delC carrier tumors to be predominantly ER+, ER+, Her2-, suggestive of good prognosis ER+ tumors or the luminal A subtype (Table 1) [34]. Also the frequency of ER+ subtypes linked to poor prognosis (ER+, PR-, Her2-; ER+, Her2+), referred to as luminal B [34], were more common for *CHEK2* mutation carriers than for non-carriers. This confirmed previous reports with regard to p.I157T [15], but was not consistent with previous reports on c.1100delC-associated tumor subtypes [47, 48]. However, the difference between our findings and these reports may have arisen from different overall cohort compositions or from differing classification methods, as the guidelines for subtype classification have changed over the years.

**Fig. 1:**
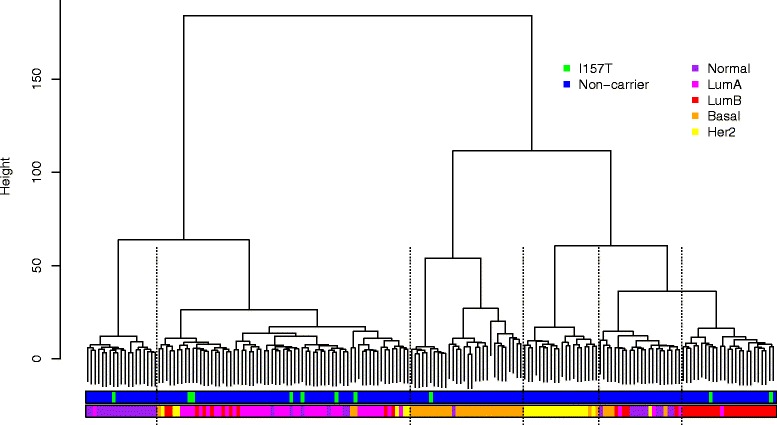
Hierarchical clustering of 183 tumor samples based on expression of the PAM50 signature genes. The *dashed lines* indicate the branch boundaries

Subtype classifications of the 183 tumors according to gene expression data and immunohistochemical markers were partly contradictory (Table 2). Similar inconsistencies between gene expression-based classification and the surrogate immunohistochemical markers have been reported previously for other data sets [40, 49]. Overall, the division between basal and luminal appeared rather consistent: only 17 (9 %) of the 183 tumors were classified differentially across the luminal-basal axis. PAM50 [32] classified three of the p.I157T carrier tumors as luminal A, two as luminal B and two as basal. Three lobular tumors were classified as normal-like (Table 2). This kind of a misclassification has been reported to be typical for lobular tumors due to their infiltrating growth pattern, which causes the tumor sample to consist of unusually high proportion of non-cancerous stromal cells [39]. St Gallen 2013 criteria classified these normal-like tumors as luminal (ER+). Furthermore, in unsupervised hierarchical clustering of the 183 tumor samples based on expression of the PAM50 signature genes (Fig. 1), two of the normal-like p.I157T tumors (HEL_045 and HEL_174) clustered within the luminal A branch suggesting that luminal A could be their true molecular subtype. In summary, luminal A appeared to be the most common subtype for p.I157T carrier tumors in the gene expression data concordantly with the findings in the BCAC data.

**Table 2:** Phenotypic classification of breast tumors from ten carriers of p.I157T

	Intrinsic subtype PAM50	IHC subtype St Gallen 2013 criteria	Histological type
HEL_045	Normal	LumA	Lobular
HEL_055	LumB	LumB	Ductal
HEL_086	LumA	LumA	Lobular
HEL_126	LumA	Basal	Ductal
HEL_128	LumA	LumB	Ductal
HEL_131	Basal	Her2pos	In situ
HEL_144	Normal	LumB	Lobular
HEL_150	Basal	LumB	Ductal
HEL_163	LumB	LumB	Ductal
HEL_174	Normal	LumA	Lobular

*IHC* immunohistochemistry, *LumB* luminal B, *LumA* luminal A, *Her2* human epidermal growth factor receptor 2

### Patient survival

P.I157T carriers had better prognosis than the c.1100delC carriers with regard to overall or breast cancer-specific survival (Table 3a and b). This difference was possibly due to the poor survival associated with c.1100delC as reported previously by several studies [13, 14, 17, 50]. No statistically significant difference in overall or breast cancer-specific survival was seen between p.I157T carriers and non-carriers. Hazard ratios in the analyses of subgroups of patients with ER-positive or lobular tumors were comparable to those of the main analyses (Table 3b and c).

**Table 3:** Risk of death or disease recurrence associated with *CHEK2*:p.I157T

(a) All breast cancer patients	Univariate analysis			Adjusted analysis		
	I157T/nc	I157T/1100delC	1100delc/nc	I157T/nc	I157T/1100delC	1100delC/nc
Early death	0.85 [0.68 - 1.07]	0.74 [0.50 - 1.09]	1.28 [1.00 - 1.64]	0.80 [0.60 - 1.07]	0.51 [0.29 - 0.90]	1.32 [0.94 - 1.86]
	*0.16*	*0.12*	*0.054*	*0.13*	*0.0190*	*0.11*
Breast cancer-specific death	0.85 [0.60 - 1.20]	0.64 [0.37 - 1.12]	1.44 [1.04 - 2.00]	0.93 [0.62 - 1.40]	0.46 [0.21 - 1.03]	1.25 [0.78 - 2.00]
	*0.36*	*0.12*	*0.030*	*0.73*	*0.058*	*0.36*
Distant metastasis relapse	1.04 [0.79 - 1.37]	0.66 [0.38 - 1.14]	1.38 [0.90 - 2.11]	1.05 [0.75 - 1.47]	0.62 [0.31 - 1.23]	1.37 [0.83 - 2.26]
	*0.79*	*0.13*	*0.14*	*0.76*	*0.17*	*0.22*
Locoregional relapse	1.43 [0.92 - 2.23]	0.81 [0.58 - 1.13]	2.07 [1.16 - 3.69]	1.62 [0.99 - 2.66]	0.91 [0.33 - 2.52]	1.26 [0.59 - 2.70]
	*0.11*	*0.21*	*0.014*	*0.056*	*0.85*	*0.55*
Second breast cancer	1.54 [0.85 - 2.78]	0.69 [0.47 - 1.03]	2.88 [1.68 - 4.98]	2.03 [1.05 - 3.92]	0.69 [0.42 - 1.13]	3.62 [1.82 - 7.21]
	*0.15*	*0.070*	*0.00015*	*0.035*	*0.14*	*0.00026*
(b) Patients with ER+ breast cancer	Univariate analysis			Adjusted analysis		
	I157T/nc	I157T/1100delC	1100delc/nc	I157T/nc	I157T/1100delC	1100delC/nc
Early death	0.81 [0.61 - 1.07]	0.62 [0.39 - 0.99]	1.32 [0.98 - 1.78]	0.77 [0.55 - 1.07]	0.46 [0.25 - 0.85]	1.52 [1.06 - 2.17]
	*0.14*	*0.044*	*0.067*	*0.12*	*0.013*	*0.022*
Breast cancer-specific death	0.80 [0.51 - 1.23]	0.47 [0.23 - 0.96]	1.46 [0.96 - 2.22]	0.80 [0.49 - 1.32]	0.33 [0.13 - 0.84]	1.50 [0.92 - 2.45]
	*0.30*	*0.038*	*0.074*	*0.39*	*0.019*	*0.10*
Distant metastasis relapse	1.00 [0.71 - 1.40]	0.55 [0.29 - 1.02]	1.58 [0.99 - 2.54]	1.03 [0.70 - 1.51]	0.56 [0.26 -1.19]	1.61 [0.94 - 2.77]
	*0.98*	*0.057*	*0.056*	*0.88*	*0.13*	*0.083*
Locoregional relapse	1.46 [0.86 - 2.47]	0.77 [0.52 - 1.14]	2.33 [1.19 - 4.57]	1.58 [0.90 - 2.79]	0.93 [0.29 - 2.98]	1.08 [0.44 - 2.66]
	*0.16*	*0.19*	*0.014*	*0.11*	*0.90*	*0.87*
Second breast cancer	1.33 [0.64 - 2.75]	0.58 [0.37 - 0.92]	4.09 [2.31 - 7.26]	1.81 [0.82 - 3.96]	0.61 [0.36 - 1.04]	4.39 [2.17 - 8.87]
	*0.44*	*0.019*	*1.4E-06*	*0.14*	*0.067*	*3.8E-05*
(c) Patients with lobular breast cancer	Univariate analysis					
	I157T/nc					
Early death	0.67 [0.39 - 1.15]					
	*0.14*					
Breast cancer-specific death	0.91 [0.46 - 1.80]					
	*0.79*					
Distant metastasis relapse	0.87 [0.48 - 1.57]					
	*0.64*					
Locoregional relapse	2.45 [0.95 - 6.34]					
	*0.065*					
Second breast cancer	1.92 [0.57 - 6.49]					
	*0.29*					

Hazard ratios with 95 % confidence intervals (in parenthesis) and *p* values (italics) are reported from comparisons of p.I157T carriers and non-carriers (nc) as well as comparisons of p.I157T carriers and c.1100delC carriers. All analyses were stratified by study. Multivariate analyses were stratified by study and age category, and adjusted for tumor grade, size, progesterone receptor and nodal status. Analyses were performed also in subgroups of (b) patients with estrogen receptor-positive tumors and (c) patients with lobular tumors

*ER* estrogen receptor

Noteworthy, the c.1100delC carriers included here were only a subset of the study subjects included in the previous report by Weischer and colleagues on survival of c.1100delC carriers of the BCAC studies [17]. This was because the individual BCAC studies, which did not provide sufficient number of p.I157T carriers, were excluded from these analyses. Thus, the lack of statistical significance in some comparisons of survival difference between c.1100delC carriers and non-carriers (Table 3a and b) probably only reflected limited power due to low number of c.1100delC carriers, since the hazard ratios were always consistent with the previous report.

The different prognoses associated with p.I157T and c.1100delC possibly reflect their difference in molecular level severity of functional consequences. Therefore, it would be tempting to assume that the prognosis of all carriers of the truncating mutations would be similar to the prognosis of c.1100delC carriers. However, a recent Polish study combining three truncating *CHEK2* founder mutations found no difference between mutation carrier and non-carrier survival [51]. Some part of the conflicting findings could be explained by different patient selection: in the Polish study all patients had been diagnosed before 50 years of age, whereas here and in Weischer et al. [17] also postmenopausal patients were included in the analyses. Another potential explanation could be mutation-specific survival effects. As the Polish study combined in the analyses three different truncating mutations, the c.1100delC specific effects could have been masked, since it is the least common of the three truncating *CHEK2* mutations in Polish population [52]. Similarly as here, the Polish study reported no significant difference in survival of the p.I157T carriers and non-carriers [51].

The hazard ratios for locoregional relapse and second breast cancer (91 % contralateral, 9 % ipsilateral) associated with p.I157T and c.1100delC were close to the mutations’ relative risk estimates of primary breast cancer (Table 3) [5, 17, 53]. The marginally significant increased risk of locoregional relapse associated with p.I157T in the adjusted analyses (hazard ratio 1.62 [0.99 - 2.66], *p* value 0.056) warrants further studies, but could merely reflect the baseline risk associated with p.I157T: some of the local recurrences could represent new cancers arising during the 10-year follow-up. The risk of locoregional relapse for c.1100delC carriers was elevated in the univariate analysis but leveled out in the adjusted analysis.

### P.I157T associated differentially expressed genes

**Fig. 2:**
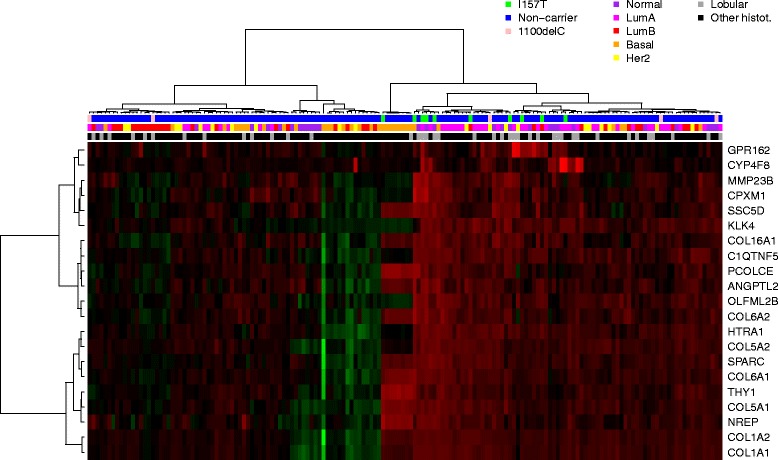
Heatmap of 21 genes expressed differentially in p.I157T carrier and non-carrier breast tumors

In order to investigate the molecular biology of p.I157T carrier tumors and to identify potential tumor-driving events and pathways, we performed an analysis of differential gene expression and subsequent functional enrichment analysis comparing ten p.I157T to 150 non-carrier tumors. We found 21 genes to be differentially expressed between p.I157T and non-carrier tumors. All of these had higher expression in the p.I157T carrier tumors (Table 4). When the 160 tumor samples were clustered according to expression of these 21 genes, the p.I157T tumors did not form a distinct cluster (Fig. 2), suggesting that high expression of these genes is not exclusive of the p.I157T mutation carrier tumors, but typical for a subgroup of breast tumors including the mutation carrier tumors. Tumors with different intrinsic subtypes appeared to be dispersed across all branches, similarly as the c.1100delC carriers suggesting that in this data set the c.1100delC carrier tumors would not be similar to the p.I157T tumors.

**Table 4:** Differentially expressed genes in breast tumors of p.I157T carriers when compared to non-carrier tumors

Gene ID	Symbol	Description	logFC	*p* value
11283	CYP4F8	Cytochrome P450, family 4, subfamily F, polypeptide 8	1.10	0.00026
1289	COL5A1	Collagen, type V, alpha 1	1.24	0.00049
1292	COL6A2	Collagen, type VI, alpha 2	0.90	0.00056
56265	CPXM1	Carboxypeptidase X (M14 family), member 1	1.04	0.00063
1277	COL1A1	Collagen, type I, alpha 1	1.25	0.0011
23452	ANGPTL2	Angiopoietin-like 2	0.85	0.0020
5118	PCOLCE	Procollagen C-endopeptidase enhancer	0.90	0.0021
284297	SSC5D	Scavenger receptor cysteine rich family, 5 domains	0.84	0.0025
25903	OLFML2B	Olfactomedin-like 2B	0.88	0.0026
27239	GPR162	G protein-coupled receptor 162	0.73	0.0026
5654	HTRA1	HtrA serine peptidase 1	0.96	0.0027
9315	NREP	Neuronal regeneration-related protein	0.78	0.0028
1278	COL1A2	Collagen, type I, alpha 2	1.02	0.0046
8510	MMP23B	Matrix metallopeptidase 23B	0.73	0.0055
114902	C1QTNF5	C1q and tumor necrosis factor-related protein 5	0.77	0.0057
6678	SPARC	Secreted protein, acidic, cysteine-rich (osteonectin)	0.88	0.0073
9622	KLK4	Kallikrein-related peptidase 4	1.03	0.0082
1291	COL6A1	Collagen, type VI, alpha 1	0.92	0.0084
1307	COL16A1	Collagen, type XVI, alpha 1	0.73	0.0090
1290	COL5A2	Collagen, type V, alpha 2	0.93	0.0095
7070	THY1	Thy-1 cell surface antigen	0.88	0.0098

*logFC* logarithm of fold change

### Enrichment of features associated with lobular breast cancer

**Fig. 3:**
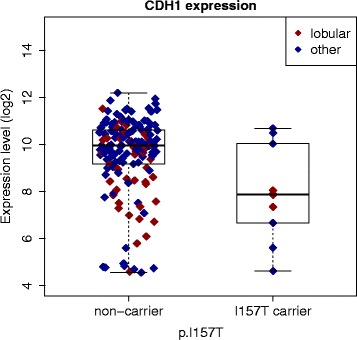
CDH1 gene expression in p.I157T carrier and non-carrier tumors

The list of 21 differentially expressed genes contained seven collagen genes (Table 4), which were a major driver in the functional enrichment analysis. The enriched annotations from DAVID [54] analysis included characteristics of the collagen family and their related functions such as ‘focal adhesion’, ‘extracellular matrix (ECM) organization’ and ‘ECM-receptor interaction’ (Additional file 1: Table S5). Similar results were obtained from the GSEA analysis (Additional file 1: Table S6, Additional file 2: Figure S1). Since collagens are usually expressed by stromal fibroblasts, the findings may suggest that infiltrating growth pattern, typical for lobular tumors [39], could be more common also for non-lobular p.I157T carrier tumors than for the non-carrier tumors. Further support for this hypothesis came from the GSEA, which showed cadherin 1 (CDH1) target genes to be significantly enriched among genes, whose expression was lower in p.I157T than in non-carrier tumors (Additional file 1: Table S6). CDH1 silencing is generally considered as a defining characteristic of lobular tumors and it is often caused by somatic mutations targeting the *CDH1* gene itself [39]. However, since the differential gene expression analysis, which was also the basis for the ranked gene list used as an input to GSEA, was adjusted for the lobular tumor type, the impact of the diagnosed lobular cancers on these findings should have been minimal. *CDH1* gene expression was lower in p.I157T carriers tumors in the adjusted analysis (log_2_ fold change -1.12, *p* value 0.03, Fig. 3), but it did not exceed the preset threshold for significance. Previously, we have reported *CDH1* mRNA expression to be higher in c.1100delC carrier than in non-carriers tumors [10]. Therefore, *CDH1* expression appears to be yet another factor, which is not shared by breast tumors from carriers of the two CHEK2 mutations, p.157 T and c.1100delC, and possibly reflects somatic changes, which have taken place during the clonal evolution of the p.I157T carrier tumors [39]. Taken together, these results suggest that besides the fact that the lobular tumors are more common among p.I157T carriers and non-carriers, the association between p.I157T and lobular features could be even stronger than what is suggested by the diagnosed histological types.

### Enrichment of cancer associated gene signatures

In the GSEA analysis, several independent MSigDB [38] gene signatures related to epithelial-to-mesenchymal transition (EMT) [55–57], stromal stem cells [58] or invasive behavior [59, 60] were enriched at the top of the gene list with higher expression in p.I157T carrier tumors than in non-carrier tumors (Additional file 1: Table S6). These observations may reflect higher stromal content of the p.I157T carrier tumor samples, as the samples were not prepared at a single cell level. However, to prevent such confounding effects the tumor sample sections were selected by an experienced breast cancer pathologist. Furthermore, the above mentioned MSigDB signatures originated from carefully designed experiments tailored to detect the true signal from cancerous epithelial cells and to escape the effects of non-cancerous stromal cells. The enrichment of these signatures may suggest that the p.I157T carrier tumors have an intrinsically invasive nature. However, this should have been reflected into poor prognosis for the p.I157T carriers, which we did not see in the survival analyses. On the other hand, it is possible that higher state of differentiation of the tumor cells suggested by low grade accompanied with the invasive nature can be seen in the prognosis only in the long run, and within the 10-year follow-up period is only reflected in the slightly elevated risk of local recurrence. All in all, these observations deserve further studies before any definitive conclusions can be made.

In addition to CDH1, tumor suppressor retinoblastoma 1 (RB1) appeared as a potential gene expression regulator, whose activity was reduced in p.I157T carrier tumors in comparison to non-carrier tumors (Additional file 1: Table S6, Additional file 1: Figure S1). RB1 and its direct downstream target E2F-1 are both targets of the CHEK2 protein [61, 62]. Thus, the differential expression of the RB1 target genes possibly reflects compromised CHEK2 function in the p.I157T carrier tumors.

Noteworthy, the two differential gene expression studies on c.1100delC carrier tumors have reported enrichment of genes of WNT and FGF pathways [10, 47], which regulate the growth and differentiation of normal breast epithelium [63–66]. Among the p.I157T-associated differentially expressed genes we did not see enrichment of any growth factor pathway. These notions on differences in gene expression signatures are more descriptive than definitive by nature, but they further emphasize intrinsic biological differences between p.I157T and 1100deC carrier tumors.

## Conclusions

Based on our analyses, breast cancers of p.I157T and c.1100delC *CHEK2* mutation carriers differ in disease severity as seen especially in differences in tumor grade and patient survival, as well as in intrinsic biological features as seen in differences in histological type and gene expression profiles. Thus, it appears that even though both mutations have been proven to compromise the protein function [6, 9, 11], they have different consequences on the disease phenotype, and prognostic findings based on one mutation cannot be generalized to the other. Furthermore, our results raise a hypothesis that the increased risk of locoregional relapse for p.I157T carriers could be caused by intrinsically invasive nature of the tumor cells. Future studies with longer follow-up are needed to test this hypothesis.

## Additional files

Additional file 1: Table S1. Description, genotyping methods and references for individual studies as reported by the studies. **Table S2.** Genotype and follow-up data availability in individual studies. **Table S3.** Sources and scoring of TP53 immunohistochemistry data used in this study. **Table S4.** Pathological characteristics of 183 breast tumors used in the gene expression analysis. **Table S5.** Functional annotations enriched in the 21 differentially expressed genes. **Table S6.** Enriched gene sets at the high and low edges of a list of 1852 genes ranked according to differences between p.I157T and non-carrier breast tumors. (DOCX 72 kb) Additional file 2: Figure S1. Enrichment plots of the high ranking gene sets from the gene set enrichment analyses (GSEA). (PDF 364 kb)

## Abbreviations

BCAC: Breast Cancer Association Consortium
CDH1: Cadherin 1
CHEK2: Checkpoint kinase 2
ECM: Extracellular matrix
EMT: Epithelial-to-mesenchymal transition
ER: Estrogen receptor
FHA: Forkhead associated
GSEA: Gene set enrichment analysis
Her2: Human epidermal growth factor receptor 2
PR: Progesterone receptor
RB1: Retinoblastoma 1
TP53: Tumor protein 53
