# Supplementary material for: Patient survival and tumor characteristics associated with CHEK2:p.I157T – findings from the Breast Cancer Association Consortium
Source: Breast Cancer Res. 2016 Oct 3;18:98. doi: 10.1186/s13058-016-0758-5 (PMC5048645; doi:10.1186/s13058-016-0758-5)
Supplement: Additional file 1: Table S1. — Description, genotyping methods and references for individual studies as reported by the studies. Table S2. Genotype and follow-up data availability in individual studies. Table S3. Sources and scoring of TP53 immunohistochemistry data used in this study. Table S4. Pathological characteristics of 183 breast tumors used in the gene expression analysis. Table S5. Functional annotations enriched in the 21 differentially expressed genes. Table S6. Enriched gene sets at the high and low edges of a list of 1852 genes ranked according to differences between p.I157T and non-carrier breast tumors. (DOCX 72 kb) [file 13058_2016_758_MOESM1_ESM.docx]

**Table S1.** Description, genotyping methods and references for individual studies as reported by the studies.

| **Study** | **Acronym** | **Country** | **Study design** | **Case definition** | **Partici-**  **pation**  **rates** | **Selected familial cases** | **Age range** | **I157T genotyping methods (genotyping year)** | **1100delC genotyping methods (genotyping year)** | **Ref.** |
| --- | --- | --- | --- | --- | --- | --- | --- | --- | --- | --- |
| Bavarian Breast Cancer Cases and Controls | BBCC | Germany | Hospital based cases | Consecutive, unselected cases with invasive breast cancer recruited at the University Breast Centre, Franconia in Northern Bavaria during 2002-2010. | 95 % | No | 22 – 96 | iPlex (2010)  iCOGs (2011) | TaqMan (2009) | [1, 2] |
| Copenhagen General Population Study | CGPS | Denmark | Population-based | Consecutive, incident cases from 1 hospital with centralized care for a population of 400,000 women from 2001 to the present. | 96 % | No | 26 – 100 | TaqMan (2010)  iCOGs (2011) | TaqMan (2009) | [3, 4] |
| Hannover Breast Cancer Study | HABCS | Germany | Hospital-based case-control study | Cases who received radiotherapy for breast cancer at Hannover Medical School between 1997-2003, unselected for age or family history | 80 % | No | 25 – 91 | Fluidigm (2010) | TaqMan (2009) | [5] |
| Helsinki Breast Cancer Study | HEBCS | Finland | Hospital-based case-control study + additional familial cases | (1) Consecutive cases (883) from the Department of Oncology, Helsinki University Central Hospital 1997-8 and 2000 (2) Consecutive cases (986) from the Department of Surgery, Helsinki University Central Hospital 2001 – 2004 (3) Familial breast cancer patients (536) from the Helsinki University Central Hospital, Departments of Oncology and Clinical Genetics (1995-) | (1) 79%  (2) 87%  (3) 90% | Subset | 22 – 96 | iPlex (2010)  iCOGs (2011) | TaqMan (2009) | [6-8] |
| Karolinska Breast Cancer Study | KARBAC | Sweden | Population and hospital-based cases | 1. Familial cases from Department of Clinical Genetics, Karolinska University Hospital, Stockholm. 2. Consecutive cases from Department of Oncology, Huddinge & Söder Hospital, Stockholm 1998-2000 | (1) NA (2) 70% | Subset | 24 – 88 | Fluidigm (2010) iCOGs (2011) | TaqMan (2009) | [9, 10] |
| Kuopio Breast Cancer Project | KBCP | Finland | Population-based prospective clinical cohort | Women seen at Kuopio University Hospital between 1990 and 1995 because of breast lump, mammographic abnormality, or other breast symptom who were found to have breast cancer | 86 % | No | 23 – 92 | iCOGs (2011) | TaqMan (2009) | [11, 12] |
| Mammary Carcinoma Risk Factor Investigation | MARIE | Germany | Population-based case-control study | Incident cases diagnosed from 2001-2005 in the study region Hamburg in Northern Germany, and from 2002-2005 in the study region Rhein-Neckar-Karlsruhe in Southern Germany. | 45,3% | No | 50 – 74 | iPlex (2010)  iCOGs (2011) |  | [13] |
| Mayo Clinic Breast Cancer Study | MCBCS | USA | Hospital-based case-control study | Incident cases residing in 6 states (MN, WI, IA, IL, ND, SD) seen at the Mayo Clinic in Rochester, MN from 2002-5 | 68,0% | No | 22 – 89 | iPlex (2010)  iCOGs (2011) | TaqMan (2009) | [14] |
| Oulu Breast Cancer Study | OBCS | Finland | Hospital-based case-control study | Consecutive incident cases diagnosed at the Oulu University Hospital between 2000 and 2004. | 71 % | No | 28 – 92 | iCOGs (2011) |  | [15] |
| Ontario Familial Breast Cancer Registry | OFBCR | Canada | Population-based familial case-control study | Incident invasive breast cancer cases diagnosed between 1 Jan 1996-31 Dec 1998 were identified from the Ontario Cancer Registry. All women between the ages of 20-54 years, a 35% random sample of those between the ages of 55-69 years, and all men between the ages of 20-79 years who were diagnosed in this period were considered eligible for enrollment in the OBCFR. During 2001-2005, enrollment was limited to minority and high-risk families. | 45 % | Subset | 22 – 81 | iPlex (2010)  iCOGs (2011) | TaqMan (2009) | [16] |
| NCI Polish Breast Cancer Study | PBCS | Poland | Population-based case-control study | Incident cases from 2000-2003 identified through a rapid identification system in participating hospitals covering ~ 90% of all eligible cases, and cancer registries in Warsaw and Łódź covering 100% of all eligible cases | 66 % | No | 27 – 75 | TaqMan (2010)  iCOGs (2011) | TaqMan (2009) | [17] |
| Karolinska Mammography Project for Risk Prediction of Breast Cancer - prevalent cases | pKARMA | Sweden | Case-control study | Incident cases from Jan 2001 – Dec 2008 from the Stockholm/Gotland area. Identified through the Stockholm breast cancer registry. | 60 % | no | 25 – 79 | iCOGs (2011) |  |  |
| Singapore and Sweden Breast Cancer Study | SASBAC | Sweden | Population-based case-control study | Incident cases from October 1993 to March 1995 identified via the 6 regional cancer registries in Sweden, to which reporting is mandatory. | 73 % | No | 50 – 74 | iPlex (2010)  iCOGs (2011) | TaqMan (2009) | [18] |
| Study of Epidemiology and Risk factors in Cancer Heredity | SEARCH | UK | Population-based case-control study | 2 groups of cases identified through East Anglian Cancer Registry; 1) prevalent cases diagnosed 1991-1996 under 55 years of age at diagnosis, recruited 1996-2002; 2) incident cases diagnosed since 1996 under 70 years of age at diagnosis, recruited 1996-present. | 64 % | No | 23 – 69 | Fluidigm (2010)  iCOGs (2011) | TaqMan (2009) | [19] |
| IHCC-Szczecin Breast Cancer Study | SZBCS | Poland | Hospital based case-control study | Prospectively ascertained cases of invasive breast cancer patients diagnosed at the Regional Oncology Hospital (Szczecin) in the years 2002, 2003, 2006 and 2007 or the University Hospital from 2002 to 2007 in Szczecin, West-Pomerania, Poland. Patients with pure intraductal or intralobular cancer were excluded (DCIS or LCIS) but patients with DCIS with micro-invasion were included. | >95% | No | 26 - 88 | TaqMan (2010)  iCOGs (2011) |  | [20, 21] |

References

1. Fasching PA, Loehberg CR, Strissel PL, Lux MP, Bani MR, Schrauder M, et al. Single nucleotide polymorphisms of the aromatase gene (CYP19A1), HER2/neu status, and prognosis in breast cancer patients. Breast Cancer Res Treat. 2008;112:89-98; doi:10.1007/s10549-007-9822-2.

2. Schrauder M, Frank S, Strissel PL, Lux MP, Bani MR, Rauh C, et al. Single nucleotide polymorphism D1853N of the ATM gene may alter the risk for breast cancer. J Cancer Res Clin Oncol. 2008;134:873-82; doi:10.1007/s00432-008-0355-9; 10.1007/s00432-008-0355-9.

3. Bojesen SE, Tybjaerg-Hansen A, Axelsson CK, Nordestgaard BG. No association of breast cancer risk with integrin beta3 (ITGB3) Leu33Pro genotype. Br J Cancer. 2005;93:167-71; doi:10.1038/sj.bjc.6602674.

4. Weischer M, Bojesen SE, Tybjaerg-Hansen A, Axelsson CK, Nordestgaard BG. Increased risk of breast cancer associated with CHEK2*1100delC. J Clin Oncol. 2007;25:57-63; doi:10.1200/JCO.2005.05.5160.

5. Dork T, Bendix R, Bremer M, Rades D, Klopper K, Nicke M, et al. Spectrum of ATM gene mutations in a hospital-based series of unselected breast cancer patients. Cancer Res. 2001;61:7608-15.

6. Syrjakoski K, Vahteristo P, Eerola H, Tamminen A, Kivinummi K, Sarantaus L, et al. Population-based study of BRCA1 and BRCA2 mutations in 1035 unselected Finnish breast cancer patients. J Natl Cancer Inst. 2000;92:1529-31.

7. Kilpivaara O, Bartkova J, Eerola H, Syrjakoski K, Vahteristo P, Lukas J, et al. Correlation of CHEK2 protein expression and c.1100delC mutation status with tumor characteristics among unselected breast cancer patients. Int J Cancer. 2005;113:575-80; doi:10.1002/ijc.20638.

8. Fagerholm R, Hofstetter B, Tommiska J, Aaltonen K, Vrtel R, Syrjakoski K, et al. NAD(P)H:quinone oxidoreductase 1 NQO1*2 genotype (P187S) is a strong prognostic and predictive factor in breast cancer. Nat Genet. 2008;40:844-53; doi:10.1038/ng.155.

9. Lindblom A, Rotstein S, Larsson C, Nordenskjold M, Iselius L. Hereditary breast cancer in Sweden: a predominance of maternally inherited cases. Breast Cancer Res Treat. 1992;24:159-65.

10. Margolin S, Werelius B, Fornander T, Lindblom A. BRCA1 mutations in a population-based study of breast cancer in Stockholm County. Genet Test. 2004;8:127-32; doi:10.1089/1090657041797365.

11. Hartikainen JM, Tuhkanen H, Kataja V, Dunning AM, Antoniou A, Smith P, et al. An autosome-wide scan for linkage disequilibrium-based association in sporadic breast cancer cases in eastern Finland: three candidate regions found. Cancer Epidemiol Biomarkers Prev. 2005;14:75-80.

12. Hartikainen JM, Tuhkanen H, Kataja V, Eskelinen M, Uusitupa M, Kosma VM, et al. Refinement of the 22q12-q13 breast cancer--associated region: evidence of TMPRSS6 as a candidate gene in an eastern Finnish population. Clin Cancer Res. 2006;12:1454-62; doi:10.1158/1078-0432.CCR-05-1417.

13. Flesch-Janys D, Slanger T, Mutschelknauss E, Kropp S, Obi N, Vettorazzi E, et al. Risk of different histological types of postmenopausal breast cancer by type and regimen of menopausal hormone therapy. Int J Cancer. 2008;123:933-41; doi:10.1002/ijc.23655; 10.1002/ijc.23655.

14. Olson JE, Ma CX, Pelleymounter LL, Schaid DJ, Pankratz VS, Vierkant RA, et al. A comprehensive examination of CYP19 variation and breast density. Cancer Epidemiol Biomarkers Prev. 2007;16:623-5; doi:10.1158/1055-9965.EPI-06-0781.

15. Erkko H, Xia B, Nikkila J, Schleutker J, Syrjakoski K, Mannermaa A, et al. A recurrent mutation in PALB2 in Finnish cancer families. Nature. 2007;446:316-9; doi:nature05609 [pii].

16. John EM, Hopper JL, Beck JC, Knight JA, Neuhausen SL, Senie RT, et al. The Breast Cancer Family Registry: an infrastructure for cooperative multinational, interdisciplinary and translational studies of the genetic epidemiology of breast cancer. Breast Cancer Res. 2004;6:R375-89; doi:10.1186/bcr801.

17. Garcia-Closas M, Egan KM, Newcomb PA, Brinton LA, Titus-Ernstoff L, Chanock S, et al. Polymorphisms in DNA double-strand break repair genes and risk of breast cancer: two population-based studies in USA and Poland, and meta-analyses. Hum Genet. 2006;119:376-88; doi:10.1007/s00439-006-0135-z.

18. Wedren S, Lovmar L, Humphreys K, Magnusson C, Melhus H, Syvanen AC, et al. Oestrogen receptor alpha gene haplotype and postmenopausal breast cancer risk: a case control study. Breast Cancer Res. 2004;6:R437-49; doi:10.1186/bcr811.

19. Lesueur F, Pharoah PD, Laing S, Ahmed S, Jordan C, Smith PL, et al. Allelic association of the human homologue of the mouse modifier Ptprj with breast cancer. Hum Mol Genet. 2005;14:2349-56; doi:10.1093/hmg/ddi237.

20. Lubinski J, Korzen M, Gorski B, Cybulski C, Debniak T, Jakubowska A, et al. Genetic contribution to all cancers: the first demonstration using the model of breast cancers from Poland stratified by age at diagnosis and tumour pathology. Breast Cancer Res Treat. 2009;114:121-6; doi:10.1007/s10549-008-9974-8; 10.1007/s10549-008-9974-8.

21. Anton-Culver H, Cohen PF, Gildea ME, Ziogas A. Characteristics of BRCA1 mutations in a population-based case series of breast and ovarian cancer. Eur J Cancer. 2000;36:1200-8.

**Table S2.** Genotype and follow-up data availability in individual studies.

|  | **I157T** | | **ch1100delc** | | **Overall survival** | | **BC specific survival** | | **DM relapse** | | **LR relapse** | | **Contralateral BC** | | **Second BC** | |
| --- | --- | --- | --- | --- | --- | --- | --- | --- | --- | --- | --- | --- | --- | --- | --- | --- |
| **study** | **nc** | **I157T** | **nc** | **1100delC** | **All** | **events** | **All** | **events** | **All** | **events** | **All** | **events** | **All** | **events** | **All** | **events** |
| **BBCC** | 1 258 | 26 | 707 | 7 | 1 310 | 207 | 1 205 | 113 | 1 159 | 113 | 1 196 | 68 | 64 | 38 | 64 | 64 |
| **CGPS** | 2 212 | 31 | 2 085 | 32 | 2 278 | 361 |  |  | 2 142 | 95 | 2 032 | 61 | 2 109 | 48 | 2 109 | 48 |
| **HABCS** | 130 | 9 | 137 | 10 | 144 | 20 | 143 | 19 |  |  |  |  | 151 | 14 | 151 | 14 |
| **HEBCS** | 1 932 | 134 | 1 966 | 80 | 2 199 | 523 | 2 168 | 294 | 2 110 | 332 | 2 137 | 114 | 2 088 | 62 | 2 088 | 62 |
| **KARBAC** | 424 | 11 | 426 | 6 | 450 | 112 | 450 | 58 | 448 | 74 | 450 | 37 | 441 | 22 | 441 | 22 |
| **KBCP** | 419 | 22 | 390 | 11 | 463 | 227 | 463 | 104 | 458 | 123 | 462 | 29 | 454 | 0 | 454 | 0 |
| **MARIE** | 2 279 | 47 |  |  | 2 302 | 286 | 2 243 | 167 | 1 608 | 212 | 1 617 | 82 | 2 244 | 26 | 2 244 | 29 |
| **MCBCS** | 1 574 | 19 | 958 | 12 | 1 621 | 120 |  |  | 115 | 78 | 118 | 35 |  |  |  |  |
| **OBCS** | 488 | 23 |  |  | 511 | 30 | 510 | 23 |  |  |  |  | 500 | 1 | 500 | 2 |
| **OFBCR** | 979 | 10 | 841 | 10 | 1 038 | 176 | 1 006 | 96 | 696 | 41 | 696 | 31 | 969 | 24 | 969 | 24 |
| **PBCS** | 1 324 | 102 | 1 418 | 6 | 1 536 | 229 | 1 370 | 58 | 1 158 | 137 | 1 044 | 23 | 1 531 | 18 | 1 531 | 18 |
| **SASBAC** | 1 187 | 18 | 1 135 | 12 | 1 220 | 200 | 1 164 | 73 | 1 156 | 19 | 1 165 | 10 | 1 180 | 4 | 1 180 | 7 |
| **SEARCH** | 6 696 | 14 | 6 422 | 85 | 6 943 | 1 070 | 6 845 | 736 |  |  |  |  | 6 338 | 86 | 6 338 | 86 |
| **SZBCS** | 276 | 29 | 181 | 0 | 305 | 29 |  |  | 156 | 58 |  |  | 281 | 1 | 281 | 1 |
| **pKARMA** | 4 318 | 95 |  |  | 4 413 | 73 | 4 413 | 47 |  |  |  |  |  |  |  |  |
| **Total** | 25 496 | 590 | 16 666 | 271 | 26 733 | 3 663 | 21 980 | 1 788 | 11 206 | 1 282 | 10 917 | 490 | 18 350 | 344 | 18 350 | 377 |

**Abbreviations:** nc: non-carriers; BC: breast cancer; DM: distant metastasis; LR: locoregional.

**Table S3.** Sources and scoring of TP53 immunohistochemistry data used in this study.

| **Study** | **stained & scored at** | **measurement** | **scoring** | **positive scores (%)** | **reference** |
| --- | --- | --- | --- | --- | --- |
| **HEBCS** | HEBCS | positive nuclei (%) | pos: > 20% positive nuclei | 22 % | [1] |
| **KBCP** | HEBCS | positive nuclei (%) | pos: > 20% positive nuclei | 18 % | - |
| **MCBCS** | HEBCS | positive nuclei (%) | pos: > 20% positive nuclei | 18 % | - |
| **OFBCR** | OFBCR | positive nuclei (%) and staining intensity | Allred's method;  pos: score > 3 (out of 8) | 33 % | [2] |
| **PBCS** | PBCS | % positive nuclei (1-100) x intensity (1-3) | pos: score > 60 (out of 300) | 18 % | [3] |
| **SEARCH** | SEARCH | staining intensity and focality | neg: no staining, or weak focal staining;  pos = strong diffuse staining | 14 % | - |

References

1. Tommiska J, Eerola H, Heinonen M, Salonen L, Kaare M, Tallila J et al. Breast cancer patients with p53 Pro72 homozygous genotype have a poorer survival. Clin Cancer Res. 2005;11:5098-103.

2. Allred DC, Harvey JM, Berardo M, Clark GM. Prognostic and predictive factors in breast cancer by immunohistochemical analysis. Mod Pathol. 1998;11:155–168.

3. Yang XR, Pfeiffer RM, Garcia-Closas M, Rimm DL, Lissowska J, Brinton LA et al. Hormonal markers in breast cancer: coexpression, relationship with pathologic characteristics, and risk factor associations in a population-based study. Cancer Res. 2007;67:10608-17.

**Table S4.** Pathological characteristics of 183 breast tumors used in the gene expression analysis. ER, PR, Her2, ki67 and TP53 statuses have been defined by immunohistochemical analysis.

|  |  | **non-carriers** | **I157T carriers** | **I157T status unknown** |
| --- | --- | --- | --- | --- |
| **ER** | negative | 36 | 2 | 7 |
|  | positive | 125 | 8 | 4 |
|  | missing | 1 | 0 | 0 |
| **PR** | negative | 57 | 5 | 8 |
|  | positive | 104 | 5 | 3 |
|  | missing | 1 | 0 | 0 |
| **Her2** | negative | 86 | 8 | 4 |
|  | positive | 19 | 1 | 0 |
|  | missing | 57 | 1 | 7 |
| **ki67** | negative | 12 | 0 | 0 |
|  | weak positive | 55 | 4 | 1 |
|  | moderate positive | 44 | 4 | 4 |
|  | strong positive | 48 | 2 | 5 |
|  | missing | 3 | 0 | 1 |
| **TP53** | negative | 102 | 9 | 4 |
|  | weak positive | 12 | 1 | 0 |
|  | moderate positive | 10 | 0 | 1 |
|  | strong positive | 30 | 0 | 4 |
|  | missing | 8 | 0 | 2 |
| **Tumor size (T)** | < 20mm | 74 | 5 | 2 |
|  | 20mm - 50mm | 76 | 5 | 8 |
|  | > 50mm | 8 | 0 | 0 |
| any size with extension to chest wall or skin; inflammatory carcinoma | | 4 | 0 | 0 |
|  | missing | 0 | 0 | 1 |
| **Lymph node status (N)** | negative | 69 | 6 | 7 |
|  | positive | 91 | 4 | 4 |
|  | missing | 2 | 0 | 0 |
| **Metastasis (M)** | negative | 156 | 8 | 9 |
|  | positive | 5 | 2 | 0 |
|  | missing | 1 | 0 | 2 |
| **Grade** | 0 | 2 | 1 | 0 |
|  | 1 | 23 | 3 | 0 |
|  | 2 | 71 | 5 | 2 |
|  | 3 | 62 | 1 | 7 |
|  | missing | 4 | 0 | 2 |
| **Histological type** | Ductal | 99 | 5 | 6 |
|  | Lobular | 41 | 4 | 1 |
|  | Other | 22 | 1 | 4 |
|  | missing | 0 | 0 | 0 |
| **Total** |  | 162 | 10 | 11 |

**Table S5.** Functional annotations enriched in the 21 differentially expressed genes.

| **Category** | **Term** | **corrected p-value** | **No genes** | **Genes** |
| --- | --- | --- | --- | --- |
| SP_PIR_KEYWORDS | trimer | 4.4E-12 | 7 | COL1A2, COL6A2, COL6A1, COL1A1, COL16A1, COL5A2, COL5A1 |
| SP_PIR_KEYWORDS | hydroxylysine | 7.0E-12 | 7 | COL1A2, COL6A2, COL6A1, COL1A1, COL16A1, COL5A2, COL5A1 |
| SP_PIR_KEYWORDS | triple helix | 7.0E-12 | 7 | COL1A2, COL6A2, COL6A1, COL1A1, COL16A1, COL5A2, COL5A1 |
| SP_PIR_KEYWORDS | hydroxyproline | 1.5E-11 | 7 | COL1A2, COL6A2, COL6A1, COL1A1, COL16A1, COL5A2, COL5A1 |
| SP_PIR_KEYWORDS | collagen | 3.2E-11 | 8 | C1QTNF5, COL1A2, COL6A2, COL6A1, COL1A1, COL16A1, COL5A2, COL5A1 |
| SP_PIR_KEYWORDS | Secreted | 4.9E-11 | 15 | OLFML2B, KLK4, SPARC, COL16A1, COL5A2, COL5A1, PCOLCE, C1QTNF5, HTRA1, CPXM1, COL1A2, COL6A2, COL6A1, COL1A1, ANGPTL2 |
| INTERPRO | IPR008160:Collagen triple helix repeat | 1.6E-10 | 8 | C1QTNF5, COL1A2, COL6A2, COL6A1, COL1A1, COL16A1, COL5A2, COL5A1 |
| SP_PIR_KEYWORDS | hydroxylation | 4.4E-10 | 7 | COL1A2, COL6A2, COL6A1, COL1A1, COL16A1, COL5A2, COL5A1 |
| GOTERM_CC_FAT | GO:0005576~extracellular region | 4.4E-09 | 17 | OLFML2B, KLK4, SPARC, COL16A1, COL5A2, COL5A1, PCOLCE, C1QTNF5, MMP23B, HTRA1, CPXM1, COL1A2, COL6A2, COL6A1, COL1A1, SSC5D, ANGPTL2 |
| SP_PIR_KEYWORDS | pyroglutamic acid | 5.5E-09 | 6 | COL1A2, COL6A2, COL6A1, COL1A1, COL5A2, COL5A1 |
| SP_PIR_KEYWORDS | extracellular matrix | 1.0E-08 | 8 | COL1A2, COL6A2, COL6A1, SPARC, COL1A1, COL16A1, COL5A2, COL5A1 |
| SP_PIR_KEYWORDS | signal | 1.1E-08 | 16 | OLFML2B, KLK4, SPARC, COL16A1, COL5A2, COL5A1, PCOLCE, THY1, C1QTNF5, HTRA1, CPXM1, COL1A2, COL6A2, COL6A1, COL1A1, ANGPTL2 |
| KEGG_PATHWAY | hsa04512:ECM-receptor interaction | 1.9E-08 | 6 | COL1A2, COL6A2, COL6A1, COL1A1, COL5A2, COL5A1 |
| SP_PIR_KEYWORDS | glycoprotein | 2.8E-08 | 17 | GPR162, OLFML2B, KLK4, SPARC, COL16A1, COL5A2, COL5A1, PCOLCE, THY1, C1QTNF5, MMP23B, CPXM1, COL1A2, COL6A2, COL6A1, COL1A1, ANGPTL2 |
| GOTERM_CC_FAT | GO:0005581~collagen | 3.6E-08 | 6 | COL1A2, COL6A1, COL1A1, COL16A1, COL5A2, COL5A1 |
| UP_SEQ_FEATURE | signal peptide | 1.4E-07 | 16 | OLFML2B, KLK4, SPARC, COL16A1, COL5A2, COL5A1, PCOLCE, THY1, C1QTNF5, HTRA1, CPXM1, COL1A2, COL6A2, COL6A1, COL1A1, ANGPTL2 |
| GOTERM_CC_FAT | GO:0005578~proteinaceous extracellular matrix | 1.5E-07 | 9 | MMP23B, COL1A2, COL6A2, COL6A1, SPARC, COL1A1, COL16A1, COL5A2, COL5A1 |
| GOTERM_CC_FAT | GO:0044421~extracellular region part | 1.6E-07 | 12 | MMP23B, HTRA1, CPXM1, COL1A2, COL6A2, COL6A1, SPARC, COL1A1, COL16A1, COL5A2, ANGPTL2, COL5A1 |
| GOTERM_CC_FAT | GO:0031012~extracellular matrix | 1.7E-07 | 9 | MMP23B, COL1A2, COL6A2, COL6A1, SPARC, COL1A1, COL16A1, COL5A2, COL5A1 |
| GOTERM_CC_FAT | GO:0044420~extracellular matrix part | 1.8E-07 | 7 | COL1A2, COL6A1, SPARC, COL1A1, COL16A1, COL5A2, COL5A1 |
| SP_PIR_KEYWORDS | Ehlers-Danlos syndrome | 7.5E-07 | 4 | COL1A2, COL1A1, COL5A2, COL5A1 |
| KEGG_PATHWAY | hsa04510:Focal adhesion | 8.0E-07 | 6 | COL1A2, COL6A2, COL6A1, COL1A1, COL5A2, COL5A1 |
| UP_SEQ_FEATURE | glycosylation site:N-linked (GlcNAc...) | 1.5E-06 | 16 | GPR162, OLFML2B, KLK4, SPARC, COL16A1, COL5A2, PCOLCE, THY1, C1QTNF5, MMP23B, CPXM1, COL1A2, COL6A2, COL6A1, COL1A1, ANGPTL2 |
| UP_SEQ_FEATURE | propeptide:C-terminal propeptide | 2.0E-06 | 4 | COL1A2, COL1A1, COL5A2, COL5A1 |
| UP_SEQ_FEATURE | domain:Fibrillar collagen NC1 | 3.0E-06 | 4 | COL1A2, COL1A1, COL5A2, COL5A1 |
| GOTERM_CC_FAT | GO:0005583~fibrillar collagen | 4.8E-06 | 4 | COL1A2, COL1A1, COL5A2, COL5A1 |
| INTERPRO | IPR000885:Fibrillar collagen, C-terminal | 5.3E-06 | 4 | COL1A2, COL1A1, COL5A2, COL5A1 |
| GOTERM_MF_FAT | GO:0048407~platelet-derived growth factor binding | 9.3E-06 | 4 | COL1A2, COL6A1, COL1A1, COL5A1 |
| SMART | SM00038:COLFI | 2.0E-05 | 4 | COL1A2, COL1A1, COL5A2, COL5A1 |
| UP_SEQ_FEATURE | region of interest:Triple-helical region | 2.1E-05 | 4 | COL6A2, COL6A1, COL1A1, COL5A1 |
| UP_SEQ_FEATURE | short sequence motif:Cell attachment site | 2.5E-05 | 5 | COL6A2, COL6A1, COL1A1, COL16A1, COL5A2 |
| GOTERM_MF_FAT | GO:0005201~extracellular matrix structural constituent | 4.9E-05 | 5 | COL1A2, COL6A2, COL1A1, COL5A2, COL5A1 |
| GOTERM_MF_FAT | GO:0019838~growth factor binding | 7.2E-05 | 5 | HTRA1, COL1A2, COL6A1, COL1A1, COL5A1 |
| PIR_SUPERFAMILY | PIRSF002255:collagen alpha 1(I) chain | 2.6E-04 | 3 | COL1A2, COL1A1, COL5A2 |
| OMIM_DISEASE | Ehlers-Danlos syndrome, type I | 4.1E-04 | 3 | COL1A1, COL5A2, COL5A1 |
| GOTERM_BP_FAT | GO:0030198~extracellular matrix organization | 4.6E-04 | 5 | COL1A2, COL6A2, COL1A1, COL5A2, COL5A1 |
| SP_PIR_KEYWORDS | cell binding | 5.2E-04 | 3 | COL6A2, COL6A1, COL16A1 |
| GOTERM_BP_FAT | GO:0043588~skin development | 8.6E-04 | 4 | COL1A2, COL1A1, COL5A2, COL5A1 |
| GOTERM_BP_FAT | GO:0030199~collagen fibril organization | 8.6E-04 | 4 | COL1A2, COL1A1, COL5A2, COL5A1 |
| SP_PIR_KEYWORDS | coiled coil | 0.0014 | 9 | OLFML2B, COL1A2, COL6A2, COL6A1, COL1A1, COL16A1, COL5A2, ANGPTL2, COL5A1 |
| GOTERM_BP_FAT | GO:0043062~extracellular structure organization | 0.0018 | 5 | COL1A2, COL6A2, COL1A1, COL5A2, COL5A1 |
| SP_PIR_KEYWORDS | disulfide bond | 0.0029 | 10 | C1QTNF5, MMP23B, OLFML2B, KLK4, CPXM1, SPARC, COL1A1, ANGPTL2, PCOLCE, THY1 |
| GOTERM_MF_FAT | GO:0005198~structural molecule activity | 0.0054 | 6 | COL1A2, COL6A2, COL1A1, COL16A1, COL5A2, COL5A1 |
| UP_SEQ_FEATURE | disulfide bond | 0.0065 | 10 | C1QTNF5, MMP23B, OLFML2B, KLK4, CPXM1, SPARC, COL1A1, ANGPTL2, PCOLCE, THY1 |
| SP_PIR_KEYWORDS | Pyrrolidone carboxylic acid | 0.0086 | 3 | COL1A2, COL1A1, THY1 |
| SP_PIR_KEYWORDS | disease mutation | 0.011 | 7 | C1QTNF5, COL1A2, COL6A2, COL6A1, COL1A1, COL5A2, COL5A1 |
| SP_PIR_KEYWORDS | skin | 0.020 | 2 | COL1A2, COL1A1 |
| GOTERM_MF_FAT | GO:0005178~integrin binding | 0.020 | 3 | COL16A1, COL5A1, THY1 |
| GOTERM_CC_FAT | GO:0005584~collagen type I | 0.020 | 2 | COL1A2, COL1A1 |
| PIR_SUPERFAMILY | PIRSF002259:collagen VI | 0.026 | 2 | COL6A2, COL6A1 |
| GOTERM_CC_FAT | GO:0005588~collagen type V | 0.027 | 2 | COL5A2, COL5A1 |
| SP_PIR_KEYWORDS | bone | 0.030 | 2 | COL1A2, COL1A1 |
| GOTERM_BP_FAT | GO:0022610~biological adhesion | 0.031 | 6 | CPXM1, COL6A2, COL6A1, COL16A1, COL5A1, THY1 |
| GOTERM_BP_FAT | GO:0008544~epidermis development | 0.035 | 4 | COL1A2, COL1A1, COL5A2, COL5A1 |
| SP_PIR_KEYWORDS | Protease | 0.037 | 4 | MMP23B, HTRA1, KLK4, CPXM1 |
| GOTERM_BP_FAT | GO:0007155~cell adhesion | 0.038 | 6 | CPXM1, COL6A2, COL6A1, COL16A1, COL5A1, THY1 |
| GOTERM_BP_FAT | GO:0007398~ectoderm development | 0.038 | 4 | COL1A2, COL1A1, COL5A2, COL5A1 |

**Note:** P-values have been corrected with the Benjamini-Hochberg method.

**Table S6.** Enriched gene sets at the high and low edges of a gene list of 1852 ranked according to differences between I157T and non-carrier breast tumors. Enrichment plots of gene sets marked with † are provided in Supplementary figure 1.

|  | **Gene set size** | **Genes in data** | **Rank at max** | **Enrichment score** | **Normalized enrichment score** | **p-value for enrichment** | **Corrected p-value** | **Gene set description** |
| --- | --- | --- | --- | --- | --- | --- | --- | --- |
| **Hallmark gene sets representing well-defined biological states or processes** | | |  |  |  |  |  |  |
| HALLMARK_EPITHELIAL_MESENCHYMAL_TRANSITION† | 200 | 105 | 183 | 0.83 | 2.02 | 0 | 0 | M5930: Genes defining epithelial-mesenchymal transition, as in wound healing, fibrosis and metastasis. |
| HALLMARK_UV_RESPONSE_DN | 144 | 33 | 150 | 0.82 | 1.71 | 0 | 0.003 | M5942: Genes down-regulated in response to ultraviolet (UV) radiation. |
| HALLMARK_MYOGENESIS | 200 | 49 | 236 | 0.74 | 1.64 | 0.003 | 0.011 | M5909: Genes involved in development of skeletal muscle (myogenesis). |
| HALLMARK_HYPOXIA | 200 | 64 | 243 | 0.68 | 1.56 | 0.004 | 0.034 | M5891: Genes up-regulated in response to low oxygen levels (hypoxia). |
| **Oncogenic signatures** |  |  |  |  |  |  |  |  |
| RB_P107_DN.V1_UP† | 140 | 38 | 180 | 0.85 | 1.84 | 0 | 0 | M2802: Genes up-regulated in primary keratinocytes from RB1 and RBL1 skin specific knockout mice. |
| **Computational gene sets from cancer-oriented microarray data** |  |  |  |  |  |  |  |  |
| MODULE_47† | 225 | 109 | 260 | 0.84 | 2.07 | 0 | 0 | M16395: Genes in the cancer module 47 -- ECM and collagens |
| MODULE_1† | 368 | 151 | 260 | 0.73 | 1.87 | 0 | 0 | M4051: Genes in the cancer module 1 -- Ovary genes |
| MODULE_105† | 200 | 49 | 160 | 0.82 | 1.85 | 0 | 0 | M6479: Genes in the cancer module 105 (cell cycle and mitosis) |
| GNF2_PTX3 | 36 | 20 | 73 | 0.88 | 1.73 | 0 | 0.016 | M11282: Neighborhood of PTX3 pentraxin-related gene, rapidly induced by IL-1 beta in the GNF2 expression compendium. |
| MODULE_122 | 141 | 56 | 160 | 0.76 | 1.71 | 0.0014 | 0.036 | M414: Genes in the cancer module 122 -- Adhesion molecules |
| **Gene ontology** |  |  |  |  |  |  |  |  |
| EXTRACELLULAR_MATRIX† | 100 | 36 | 180 | 0.87 | 1.87 | 0 | 0 | M18403: Genes annotated by the GO term GO:0031012. A structure lying external to one or more cells, which provides structural support for cells or tissues; may be completely external to the cell (as in animals) or be part of the cell (as in plants). |
| PROTEINACEOUS_EXTRACELLULAR_MATRIX | 98 | 36 | 180 | 0.87 | 1.86 | 0 | 0 | M15654: Genes annotated by the GO term GO:0005578. A layer consisting mainly of proteins (especially collagen) and glycosaminoglycans (mostly as proteoglycans) that forms a sheet underlying or overlying cells such as endothelial and epithelial cells. The proteins are secreted by cells in the vicinity. |
| EXTRACELLULAR_MATRIX_PART | 57 | 16 | 115 | 0.92 | 1.74 | 0 | 0.0039 | M476: Genes annotated by the GO term GO:0044420. Any constituent part of the extracellular matrix, the structure lying external to one or more cells, which provides structural support for cells or tissues; may be completely external to the cell (as in animals) or be part of the cell (as often seen in plants). |
| EXTRACELLULAR_REGION_PART | 338 | 103 | 180 | 0.70 | 1.70 | 0 | 0.015 | M18601: Genes annotated by the GO term GO:0044421. Any constituent part of the extracellular region, the space external to the outermost structure of a cell. For cells without external protective or external encapsulating structures this refers to space outside of the plasma membrane. This term covers constituent parts of the host cell environment outside an intracellular parasite. |
| EXTRACELLULAR_REGION | 447 | 139 | 180 | 0.66 | 1.68 | 0 | 0.021 | M16255: Genes annotated by the GO term GO:0005576. The space external to the outermost structure of a cell. For cells without external protective or external encapsulating structures this refers to space outside of the plasma membrane. This term covers the host cell environment outside an intracellular parasite. |
| ENDOPEPTIDASE_ACTIVITY | 117 | 19 | 181 | 0.86 | 1.67 | 0 | 0.021 | M6078: Genes annotated by the GO term GO:0004175. Catalysis of the hydrolysis of nonterminal peptide linkages in oligopeptides or polypeptides, and comprising any enzyme of sub-subclasses EC:3.4.21-99. They are classified according to the presence of essential catalytic residues or ions at their active sites. |
| **Curated gene sets of canonical pathways** |  |  |  |  |  |  |  |  |
| NABA_CORE_MATRISOME† | 275 | 98 | 183 | 0.84 | 2.06 | 0 | 0 | M5884: Ensemble of genes encoding core extracellular matrix including ECM glycoproteins, collagens and proteoglycans. |
| REACTOME_AXON_GUIDANCE | 251 | 36 | 24 | 0.90 | 1.93 | 0 | 0 | M8821: Genes involved in Axon guidance. |
| NABA_ECM_GLYCOPROTEINS | 196 | 69 | 243 | 0.79 | 1.90 | 0 | 0 | M3008: Genes encoding structural ECM glycoproteins. |
| REACTOME_SIGNALING_BY_PDGF† | 122 | 25 | 33 | 0.93 | 1.90 | 0 | 0 | M2049: Genes involved in Signaling by PDGF. |
| REACTOME_DEVELOPMENTAL_BIOLOGY | 396 | 50 | 24 | 0.85 | 1.88 | 0 | 0 | M509: Genes involved in Developmental Biology. |
| KEGG_FOCAL_ADHESION† | 201 | 40 | 78 | 0.86 | 1.86 | 0 | 0 | M7253: Focal adhesion. |
| NABA_MATRISOME | 1028 | 256 | 234 | 0.71 | 1.85 | 0 | 0 | M5889: Ensemble of genes encoding extracellular matrix and extracellular matrix-associated proteins. |
| PID_INTEGRIN1_PATHWAY | 66 | 23 | 112 | 0.92 | 1.84 | 0 | 0 | M18: Beta1 integrin cell surface interactions. |
| KEGG_ECM_RECEPTOR_INTERACTION | 84 | 24 | 27 | 0.91 | 1.83 | 0 | 0 | M7098: ECM-receptor interaction. |
| PID_AVB3_INTEGRIN_PATHWAY† | 75 | 21 | 115 | 0.93 | 1.82 | 0 | 0 | M160: Integrins in angiogenesis. |
| REACTOME_EXTRACELLULAR_MATRIX_ORGANIZATION | 87 | 25 | 126 | 0.90 | 1.80 | 0 | 8.2E-05 | M610: Genes involved in Extracellular matrix organization. |
| PID_SYNDECAN_1_PATHWAY | 46 | 20 | 115 | 0.90 | 1.77 | 0 | 4.3E-04 | M198: Syndecan-1-mediated signaling events. |
| REACTOME_COLLAGEN_FORMATION | 58 | 17 | 115 | 0.93 | 1.75 | 0 | 5.2E-04 | M631: Genes involved in Collagen formation. |
| NABA_COLLAGENS | 15 | 15 | 115 | 0.93 | 1.71 | 0 | 1.9E-03 | M3005: Genes encoding collagen proteins. |
| KEGG_DRUG_METABOLISM_CYTOCHROME_P450Y† | 72 | 23 | 50 | -0.77 | -1.75 | 0 | 0.045 | M9257: Drug metabolism - cytochrome P450. |
| **Gene sets representing expression signatures of genetic and chemical perturbations** | | |  |  |  |  |  |  |
| SCHUETZ_BREAST_CANCER_DUCTAL_INVASIVE_UP† | 351 | 146 | 216 | 0.82 | 2.07 | 0 | 0 | M17471: Genes up-regulated in invasive ductal carcinoma (IDC) relative to ductal carcinoma in situ (DCIS, non-invasive). |
| ANASTASSIOU_CANCER_MESENCHYMAL_TRANSITION_SIGNATURE† | 64 | 47 | 160 | 0.91 | 2.02 | 0 | 0 | M2572: Genes in the 'mesenchymal transition signature' common to all invasive cancer types. |
| BOQUEST_STEM_CELL_UP† | 260 | 118 | 226 | 0.80 | 1.99 | 0 | 0 | M1834: Genes up-regulated in freshly isolated CD31- (stromal stem cells from adipose tissue) versus the CD31+ (non-stem) counterparts. |
| REN_ALVEOLAR_RHABDOMYOSARCOMA_DN | 408 | 92 | 164 | 0.81 | 1.96 | 0 | 0 | M19541: Genes commonly down-regulated in human alveolar rhabdomyosarcoma (ARMS) and its mouse model overexpressing PAX3-FOXO1 fusion. |
| CHICAS_RB1_TARGETS_CONFLUENT† | 567 | 166 | 188 | 0.74 | 1.90 | 0 | 0 | M2129: Genes up-regulated in confluent IMR90 cells (fibroblast) after knockdown of RB1 by RNAi. |
| ONDER_CDH1_TARGETS_2_UP† | 256 | 97 | 276 | 0.77 | 1.89 | 0 | 0 | M13867: Genes up-regulated in HMLE cells (immortalized non-transformed mammary epithelium) after E-cadhedrin (CDH1) knockdown by RNAi. |
| TURASHVILI_BREAST_LOBULAR_CARCINOMA_VS_LOBULAR_NORMAL_DN | 74 | 38 | 192 | 0.86 | 1.86 | 0 | 0 | M13547: Genes down-regulated in lobular carcinoma vs normal lobular breast cells. |
| KINSEY_TARGETS_OF_EWSR1_FLII_FUSION_DN | 329 | 86 | 168 | 0.77 | 1.86 | 0 | 0 | M13206: Genes down-regulated in TC71 and EWS502 cells (Ewing's sarcoma) by EWSR1-FLI1 as inferred from RNAi knockdown of this fusion protein. |
| SERVITJA_ISLET_HNF1A_TARGETS_UP† | 163 | 57 | 130 | 0.82 | 1.85 | 0 | 0 | M2395: Genes up-regulated in pancreatic islets upon knockout of HNF1A. |
| LANDIS_BREAST_CANCER_PROGRESSION_DN | 70 | 29 | 89 | 0.87 | 1.84 | 0 | 0 | M9908: Genes down-regulated in preneoplastic mammary tissues and whose expression is maintained in tumors. |
| TURASHVILI_BREAST_LOBULAR_CARCINOMA_VS_DUCTAL_NORMAL_UP | 69 | 34 | 160 | 0.88 | 1.84 | 0 | 0 | M10165: Genes up-regulated in lobular carcinoma vs normal ductal breast cells. |
| LANDIS_ERBB2_BREAST_TUMORS_324_DN | 149 | 49 | 143 | 0.80 | 1.82 | 0 | 0.001 | M8901: Down-regulated genes from the 324 genes identified by two analytical methods as changed in the mammary tumors induced by transgenic expression of ERBB2. |
| SUNG_METASTASIS_STROMA_UP | 110 | 38 | 91 | 0.84 | 1.82 | 0 | 0.001 | M9483: Genes up-regulated in metastatic vs non-metastatic stromal cells originated from either bone or prostate tissues. |
| CHARAFE_BREAST_CANCER_BASAL_VS_MESENCHYMAL_UP† | 121 | 45 | 429 | -0.74 | -1.96 | 0 | 0.001 | M12795: Genes up-regulated in basal-like breast cancer cell lines as compared to the mesenchymal-like ones. |
| DOUGLAS_BMI1_TARGETS_UP | 566 | 77 | 127 | 0.77 | 1.81 | 0 | 0.005 | M15103: Genes up-regulated in A4573 cells (Ewing's sarcoma, ESFT) after knockdown of BMI1 by RNAi. |
| SENGUPTA_NASOPHARYNGEAL_CARCINOMA_UP | 294 | 78 | 157 | 0.76 | 1.81 | 0 | 0.005 | M19875: Genes up-regulated in nsopharyngeal carcinoma relative to the normal tissue. |
| JECHLINGER_EPITHELIAL_TO_MESENCHYMAL_TRANSITION_UP | 71 | 31 | 70 | 0.86 | 1.81 | 0 | 0.006 | M1406: Genes up-regulated during epithelial to mesenchymal transition (EMT) induced by TGFB1 in the EpH4 cells (mammary epithelium cell line transformed by HRAS). |
| TURASHVILI_BREAST_DUCTAL_CARCINOMA_VS_LOBULAR_NORMAL_DN | 69 | 38 | 175 | -0.76 | -1.91 | 0 | 0.008 | M5618: Genes down-regulated in ductal carcinoma vs normal lobular breast cells. |
| CHEN_METABOLIC_SYNDROM_NETWORK | 1210 | 236 | 187 | 0.69 | 1.80 | 0 | 0.009 | M1920: Genes forming the macrophage-enriched metabolic network (MEMN) claimed to have a causal relationship with the metabolic syndrom traits. |
| ONDER_CDH1_TARGETS_2_DN | 464 | 150 | 426 | -0.60 | -1.89 | 0 | 0.009 | M4306: Genes down-regulated in HMLE cells (immortalized nontransformed mammary epithelium) after E-cadhedrin (CDH1) knockdown by RNAi. |
| ZHU_CMV_ALL_DN | 128 | 47 | 234 | 0.81 | 1.80 | 0 | 0.015 | M14555: Down-regulated at any timepoint following infection of primary human foreskin fibroblasts with CMV. |
| CHARAFE_BREAST_CANCER_LUMINAL_VS_MESENCHYMAL_DN | 460 | 112 | 250 | 0.73 | 1.79 | 0 | 0.015 | M9192: Genes down-regulated in luminal-like breast cancer cell lines compared to the mesenchymal-like ones. |
| GU_PDEF_TARGETS_UP | 71 | 25 | 90 | 0.88 | 1.79 | 0 | 0.020 | M3955: Integrin, VEGF, Wnt and TGFbeta signaling pathway genes up-regulated in PC-3 cells (prostate cancer) after knockdown of PDEF by RNAi. |
| SENESE_HDAC1_TARGETS_DN | 260 | 53 | 143 | 0.78 | 1.79 | 0 | 0.023 | M6100: Genes down-regulated in U2OS cells (osteosarcoma) upon knockdown of HDAC1 by RNAi. |
| PICCALUGA_ANGIOIMMUNOBLASTIC_LYMPHOMA_UP | 205 | 89 | 260 | 0.75 | 1.78 | 0 | 0.028 | M12225: Up-regulated genes in angioimmunoblastic lymphoma (AILT) compared to normal T lymphocytes. |
| TURASHVILI_BREAST_DUCTAL_CARCINOMA_VS_DUCTAL_NORMAL_DN | 198 | 86 | 213 | -0.63 | -1.86 | 0 | 0.028 | M14134: Genes down-regulated in ductal carcinoma vs normal ductal breast cells. |
| CROONQUIST_NRAS_VS_STROMAL_STIMULATION_DN | 99 | 53 | 165 | 0.79 | 1.78 | 0 | 0.036 | M4491: Genes down-regulated in ANBL-6 cell line (multiple myeloma, MM) expressing an activated form of NRAS off a plasmid vector compared to those co-cultured with bone marrow stromal cells. |
| IGLESIAS_E2F_TARGETS_UP | 60 | 60 | 79 | 0.78 | 1.78 | 0 | 0.036 | M1484: Genes up-regulated in pancreatic cells from mice with double knockout of E2F1 and E2F2 compared to wild type. |

Color coding of highlighted cells:

| EMT associated signatures | RB1 target signatures | CDH1 target signatures |
| --- | --- | --- |
| Signatures associated with extracellular matrix components | | Breast cancer histotype signatures |
