# Supplementary figures and images for: Patient survival and tumor characteristics associated with CHEK2:p.I157T – findings from the Breast Cancer Association Consortium
Source: Breast Cancer Res. 2016 Oct 3;18:98. doi: 10.1186/s13058-016-0758-5 (PMC5048645; doi:10.1186/s13058-016-0758-5)

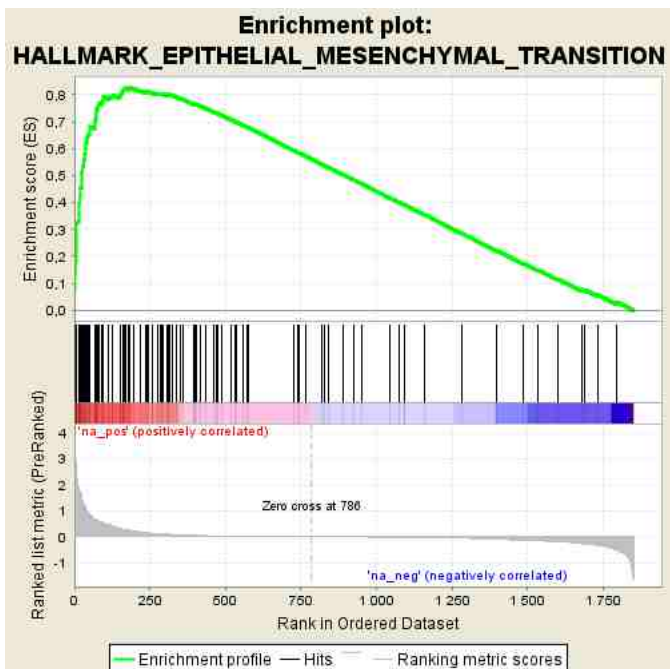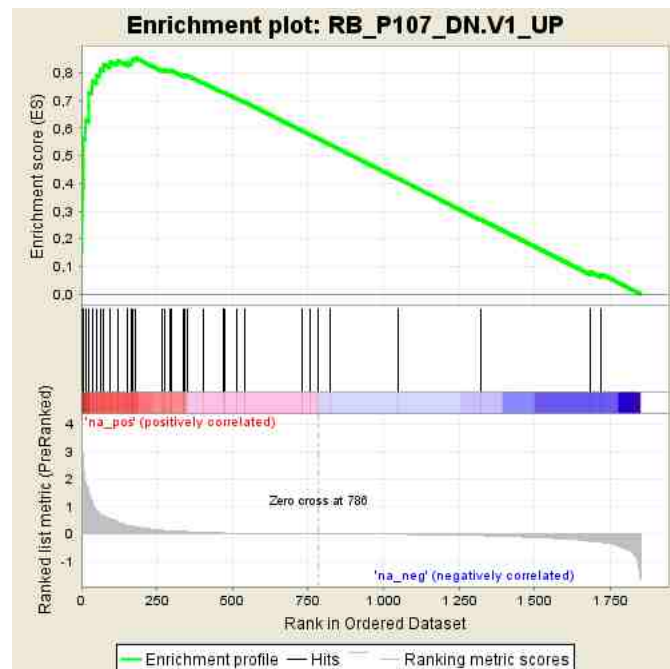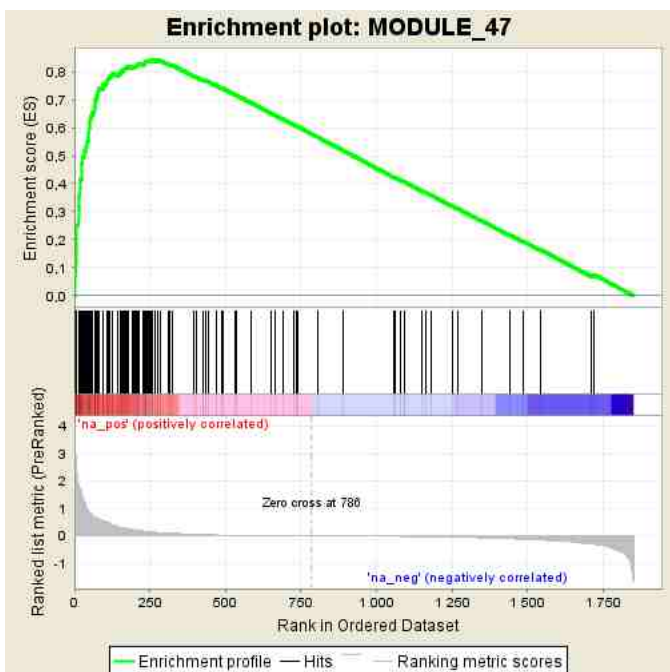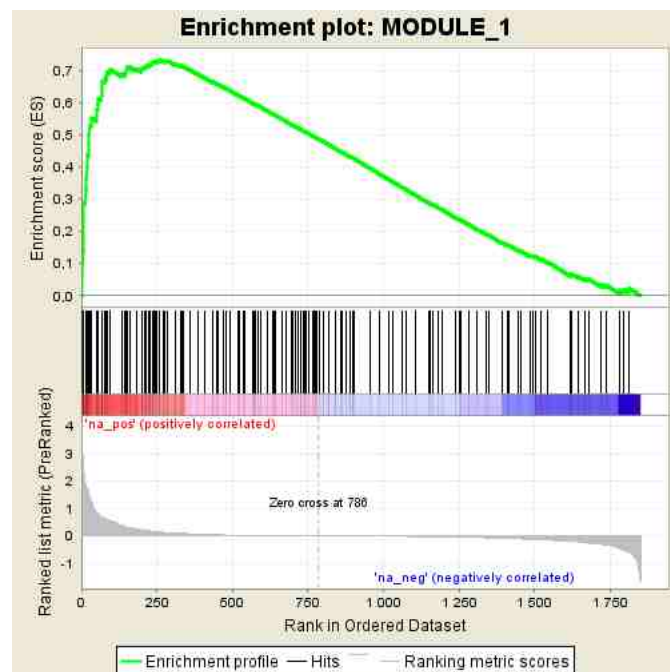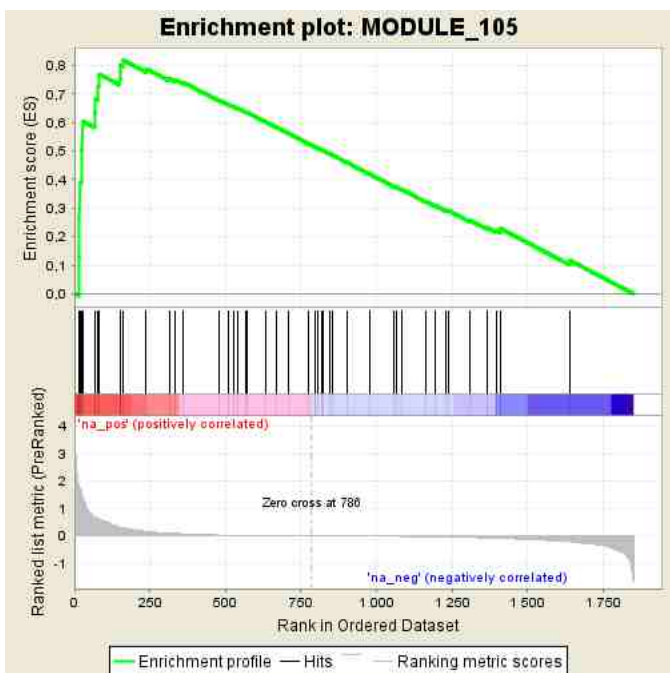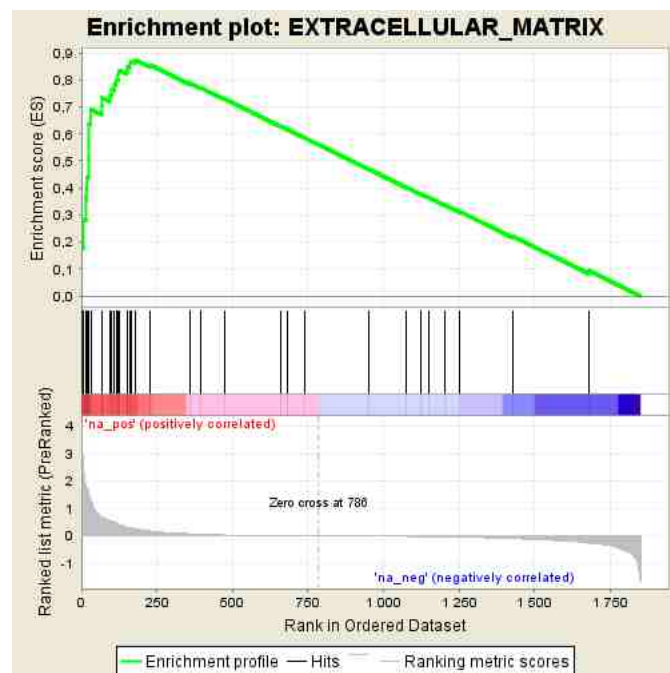

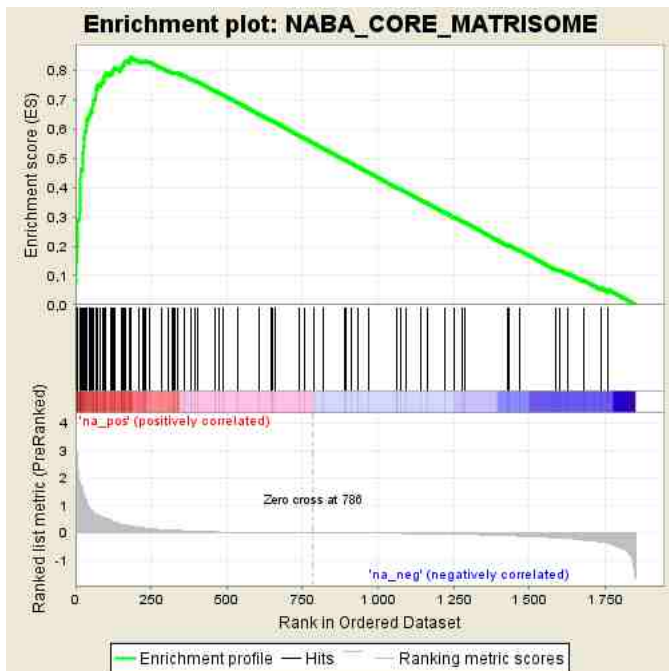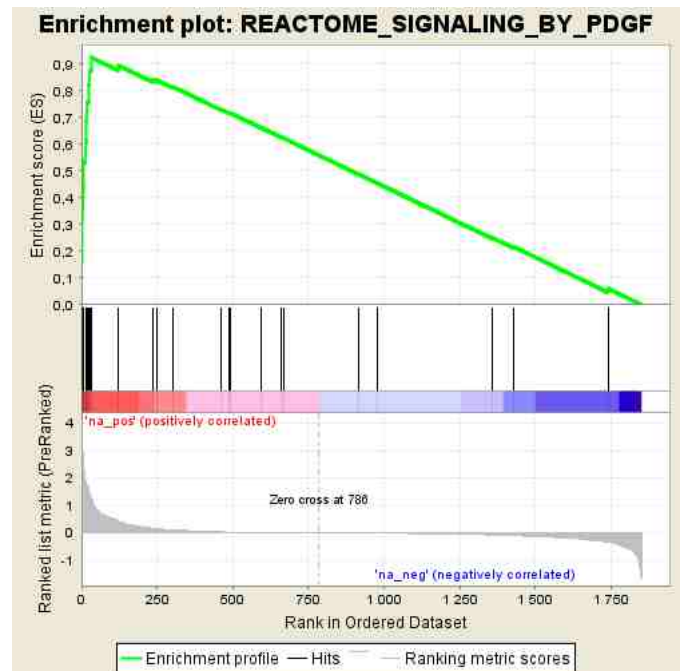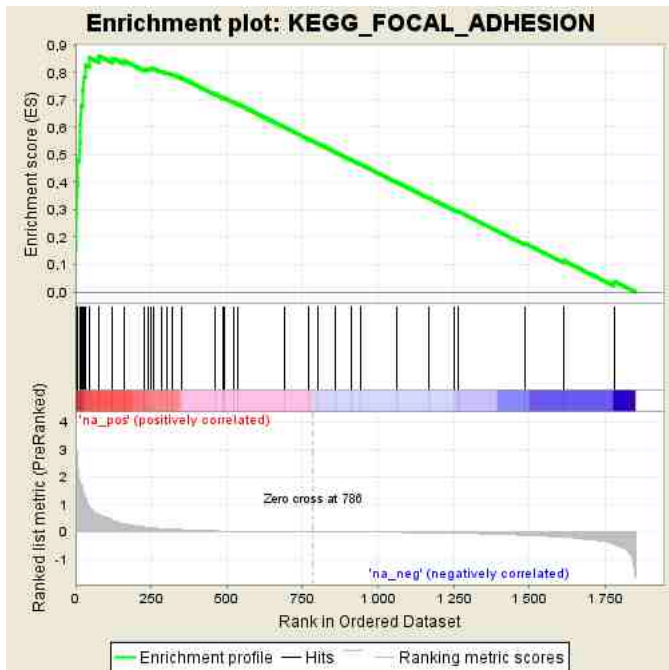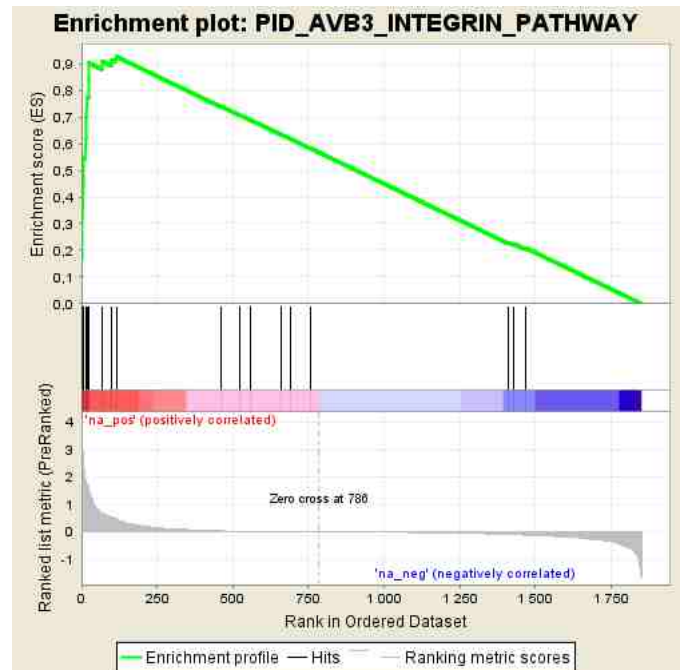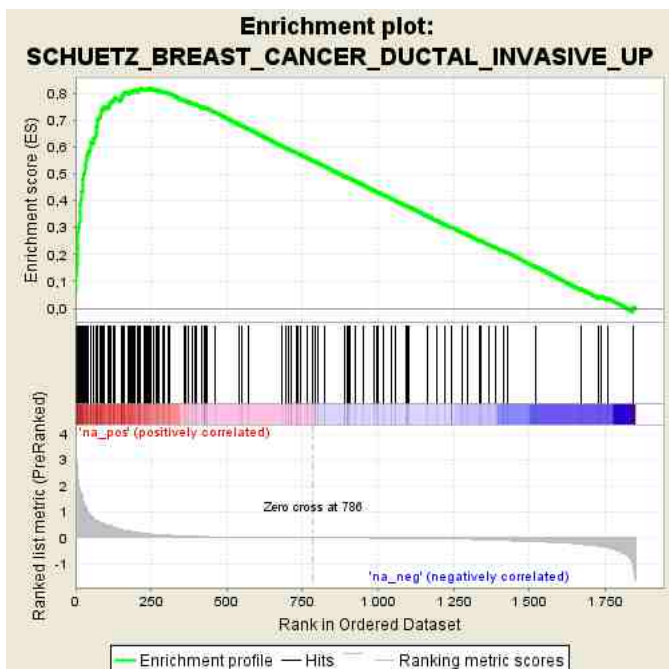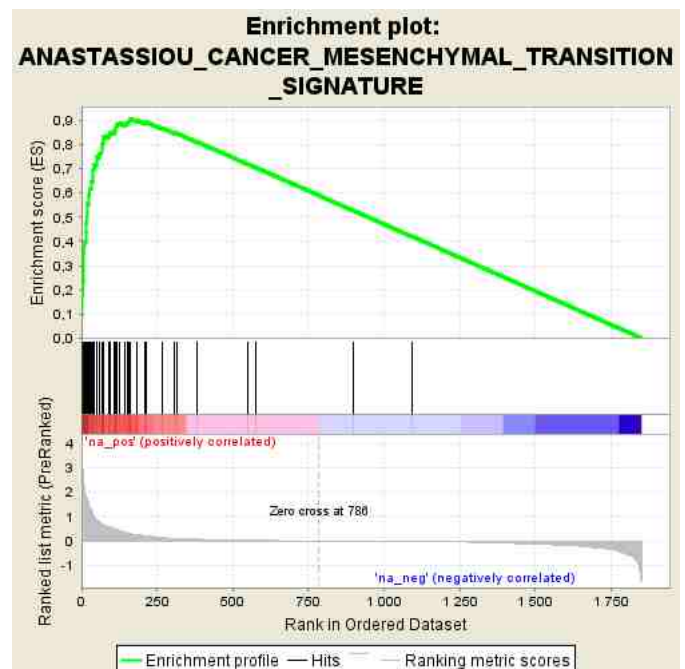

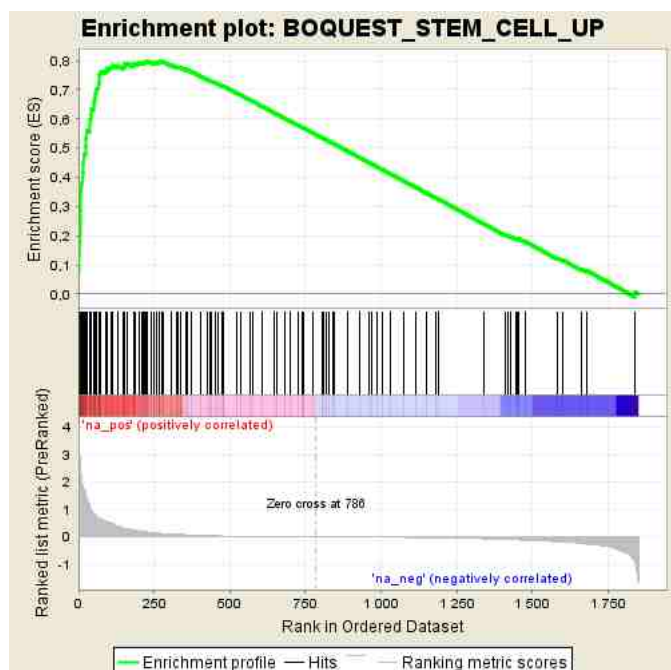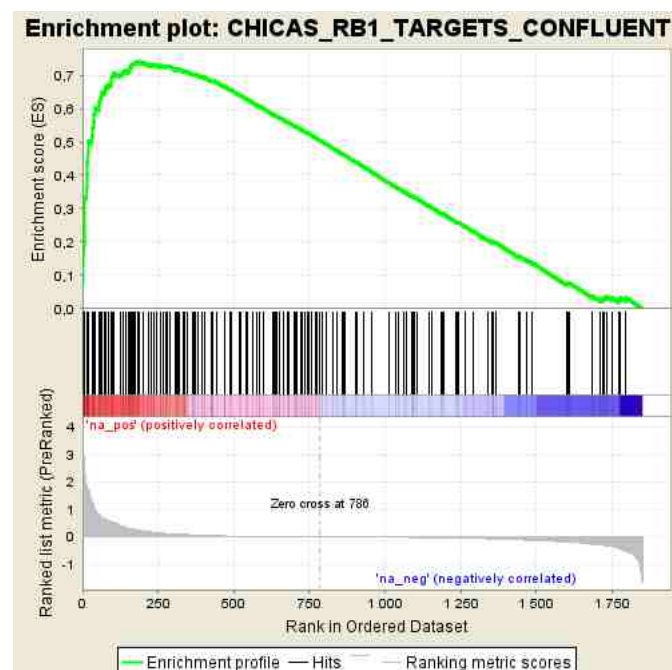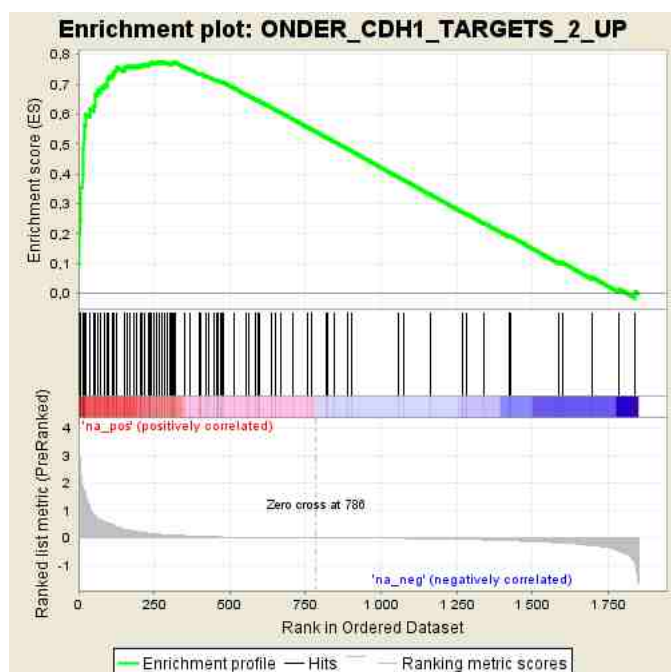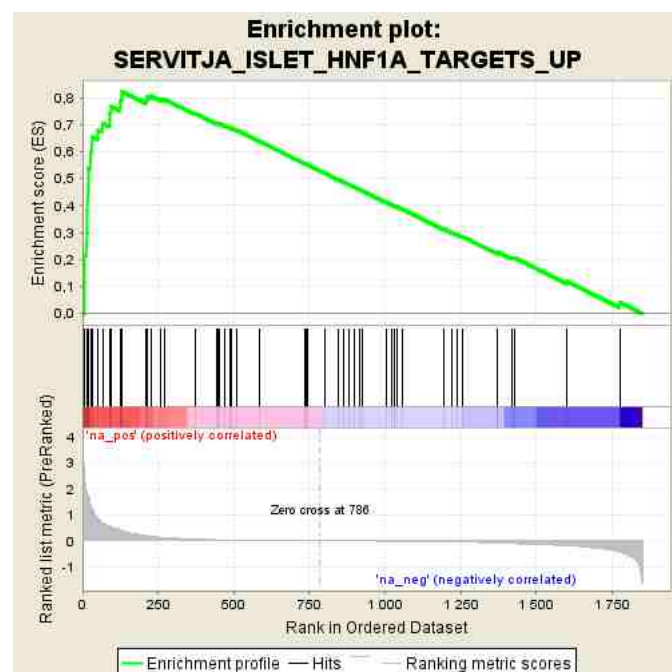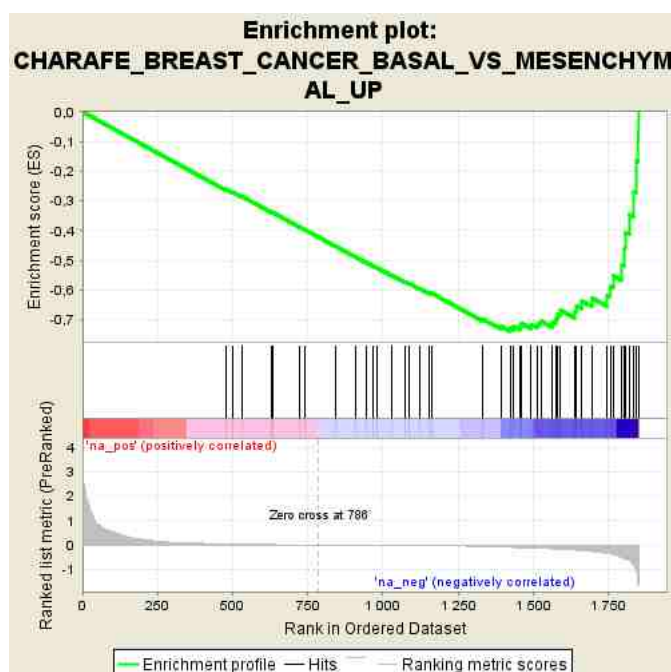

Supplement: Additional file 2: Figure S1. — Enrichment plots of the high ranking gene sets from the gene set enrichment analyses (GSEA). (PDF 364 kb) [file 13058_2016_758_MOESM2_ESM.pdf]
